# Associations of risk factor burden and genetic predisposition with the 10-year risk of atrial fibrillation: observations from a large prospective study of 348,904 participants

**DOI:** 10.1186/s12916-023-02798-7

**Published:** 2023-03-08

**Authors:** Junguo Zhang, Ge Chen, ChongJian Wang, Xiaojie Wang, Zhengmin (Min) Qian, Miao Cai, Michael G. Vaughn, Elizabeth Bingheim, Haitao Li, Yanhui Gao, Gregory Y. H. Lip, Hualiang Lin

**Affiliations:** 1 Department of Epidemiology, School of Public Health, Sun Yat-sen University, Guangzhou, 510080 China; 2 Department of Epidemiology and Biostatistics, College of Public Health, Zhengzhou University, Zhengzhou, 450001 China; 3 Department of Epidemiology and Biostatistics, College for Public Health & Social Justice, Saint Louis University, Saint Louis, MO 63103 USA; 4 School of Social Work, Saint Louis University, Saint Louis, MO 63103 USA; 5 Department of Social Medicine and Health Service Management, Health Science Center, Shenzhen University, Shenzhen, 518055 China; 6 Department of Medical Statistics, School of Basic Medicine and Public Health, Jinan University, Guangzhou, 510632 China; 7 Department of Epidemiology and Health Statistics, School of Public Health, Guangdong Pharmaceutical University, Guangzhou, 510310 China; 8 Liverpool Centre for Cardiovascular Science, University of Liverpool, Liverpool John Moores University and Liverpool Heart & Chest Hospital, Liverpool, L7 8TX UK; 9 Department of Clinical Medicine, Aalborg University, Sdr. Skovvej 15, 9000 Aalborg, Denmark

**Keywords:** Risk factor burden, Genetic predisposition, Atrial fibrillation, 10-year risk

## Abstract

**Background:**

Understanding the effects of risk factor burden and genetic predisposition on the long-term risk of atrial fibrillation (AF) is important to improve public health initiatives. However, the 10-year risk of AF considering risk factor burden and genetic predisposition is unknown.

**Methods:**

A total of 348,904 genetically unrelated participants without AF at baseline from the UK were categorized into three groups: index ages 45 years (*n* = 84,206), 55 years (*n*=117,520), and 65 years (*n*=147,178). Optimal, borderline, or elevated risk factor burden was determined by body mass index, blood pressure, diabetes mellitus, alcohol consumption, smoking status, and history of myocardial infarction or heart failure. Genetic predisposition was estimated using the polygenic risk score (PRS), constructed using 165 predefined genetic risk variants. The combined effects of risk factor burden and PRS on the risk of incident AF in 10 years were estimated for each index age. Fine and Gray models were developed to predict the 10-year risk of AF.

**Results:**

The overall 10-year risk of AF was 0.67% (95% CI: 0.61–0.73%) for index age 45 years, 2.05% (95% CI: 1.96–2.13%) for index age 55 years, and 6.34% (95% CI: 6.21–6.46%) for index age 65 years, respectively. An optimal risk factor burden was associated with later AF onset regardless of genetic predisposition and sex (*P* < 0.001). Significant synergistic interactions were observed for risk factor burden with PRS at each index age (*P* < 0.05). Participants with an elevated risk factor burden and high PRS had the highest 10-year risk of AF in reference to those who had both an optimal risk factor burden and a low PRS. At younger ages, optimal risk burden and high PRS might also lead to later onset of AF, compared to the joint effect of elevated risk burden and low/intermediate PRS.

**Conclusions:**

Risk factor burden together with a genetic predisposition is associated with the 10-year risk of AF. Our results may be helpful in selecting high-risk individuals for primary prevention of AF and facilitating subsequent health interventions.

**Supplementary Information:**

The online version contains supplementary material available at 10.1186/s12916-023-02798-7.

## Background

Atrial fibrillation (AF) is one of the most common types of cardiac arrhythmias and poses a substantial health and economic burden worldwide [1]. Along with the population aging, a rapid increase in AF prevalence and incidence has been observed [2]. Given that one third of the total AF population is asymptomatic [3], and there is a high mortality risk at the first (20%) and fifth (50%) year after an AF diagnosis, it is critical to identify high-risk population groups at risk of developing AF who are more likely to benefit from preventive interventions [4].

The European Society of Cardiology has advocated the use of risk prediction models to determine individuals who could benefit from specific preventive treatment, thus improving cost-effectiveness and enhancing healthcare [5]. Additionally, estimating individual risk over a 10-year period is the most commonly used risk model of primary prevention [6]. However, both the 10-year risk of AF developed by the Framingham Heart Study [7] and the CHARGE-AF consortium short-term risk tool [8] were developed more than a decade ago, which might not be systematically ‘recalibrated’ to contemporary AF rates. Furthermore, considering the change in the prevalence of risk factors for AF and the failure to include genetic information, these conventional risk scores might not be able to accurately represent the current impact on AF [9]. The 10-year risk developed from recent data will aid in identifying susceptible populations with appropriately predicted risk probability [10].

The estimate of the 10-year risk of AF should integrate information on widely available, easy to measure, and conventional risk factors. In addition to aging, several modifiable risk factors for AF prevention have been well established and described, including lifestyle risk factors and concomitant cardiovascular diseases [11–13]. These risk factors, whether at borderline or elevated levels, have the potential to increase the risk of AF, especially in conjunction with one another [14]. Moreover, the occurrence of AF is also affected by genetic predisposition, with approximately 11% of the variation in AF explained by total genome-wide genetic variation [15]. While genetic predisposition is often assumed to be deterministic, there is evidence that risk factor burden can attenuate high genetic risk [16, 17]. Integrating both modifiable risk factors and genetic predisposition could help improve AF risk prediction and result in more effective strategies [18]. However, standing in the way of this goal is a lack of evidence on the relationship between these two components and their association with the short-term probability of developing AF.

Leveraging data from the UK Biobank, which covers a wide range of modifiable risk factors as well as genetic data, the primary aim of this study is to estimate the 10-year risk of AF in various subgroups with different genetic and clinical factors, and second, to evaluate the combined effects and potential interactions of risk factors and genetic predisposition on AF incidence.

## Methods

### Study design and population

Data from the UK Biobank (Application Number: 69550) were applied for the present analysis. We included all participants aged 40–69 years enrolled in 2006 to 2010 with complete baseline assessments, including information on demographic and socioeconomic factors. The detailed UK Biobank protocol is available elsewhere [19].

**Fig. 1:**
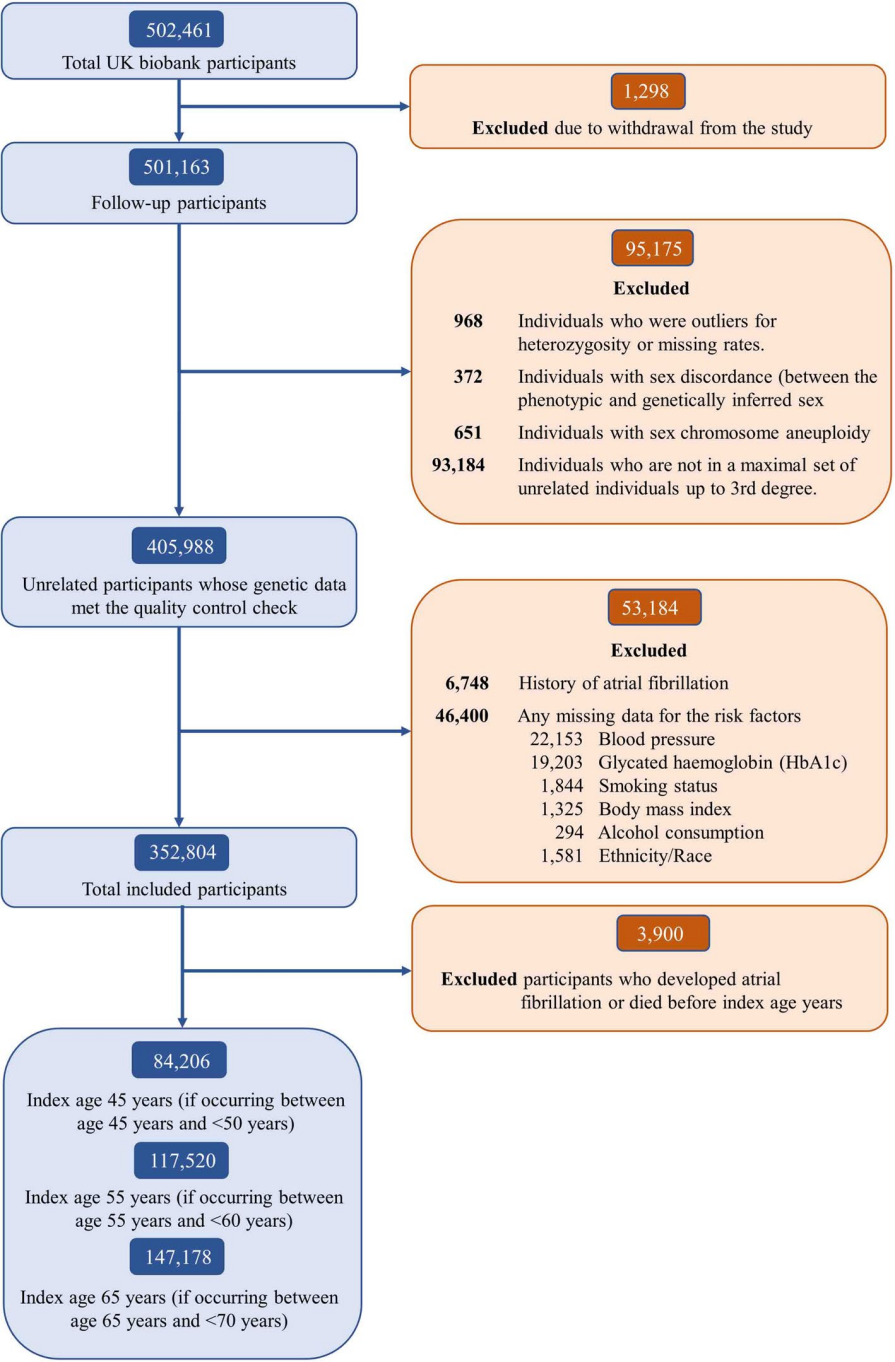
Selection of the study sample with index age years

Participants who were diagnosed with AF at baseline (*n*=6748), had missing data for the risk factors of interest (*n*=46,400, mainly due to missing blood pressure and glycated hemoglobin), missing quality-controlled genotyping data (*n*=95,175), or withdrew from the study (*n*=1298) were excluded from the current analysis. The resulting sample included 352,804 participants (Fig. 1). The detailed information on quality-controlled genotyping data was presented in Additional file 1: Text S1.

Participants were divided into three categories: index ages 45, 55, and 65 years, according to the ages at assessment: 40–49 (*n*=84,506), 50–59 (*n*=117,520), and 60–69 (*n*=147,178) years. For example, at index age 55 years, the criteria for selecting the participants were that they did not develop AF and were still alive before 55 years old. If participants were recruited between 50 and 55 years old or between 55 and 59 years old, the follow-up times of those started at 55 years or at the specific age when participant attained, respectively. The same approach was applied for participants with index ages 45 years and 65 years.

### Follow-up and outcome ascertainment

Participants free of AF were followed up from the later dates of their index age until the first AF occurrence, death, loss to follow-up, end of the ten-year follow-up, or March 31, 2021, whichever occurred first.

The outcome of the present study was the incidence of AF including both atrial fibrillation and flutter. Physician diagnosis of incident AF occurring during the 10-year follow-up was identified through the primary care system, hospital inpatient records, and death registry [19]. The date of death was ascertained by linking to death registries of the National Health Service Information Centre. Ascertainment of AF in the UK Biobank (ICD code I48) was mainly extracted from the “first occurrence” of health outcomes, which incorporates the diagnoses of AF at different time points from recruitment. We also collected the cases directly from the following codes: (1) non-cancer illness code, self-reported (1471, 1483); (2) Operation code (1524), (3) diagnoses – main/secondary ICD10 (I48, I48.0–4, I48.9); (4) underlying (primary/secondary) cause of death: ICD10 (I48, I48.0–4, I48.9); (5) diagnoses – main/secondary ICD9 (4273); (6) operative procedures – main/secondary OPCS (K57.1, K62.1–4).

### Definition of risk factors burden and profiles

In the current study, risk factor burden for each participant was constructed using a series of risk factors for AF, which was in accordance with the widely used CHARGE-AF risk score and previous literature [7, 20, 21]. The risk factors under analysis include BMI, alcohol consumption, smoking status, blood pressure, diabetes mellitus, and history of myocardial infarction or heart failure. Each risk factor was classified into two (optimal and elevated) or three (elevated, borderline, or optimal) categories, as described in Table 1. Details of the assessment of covariates are shown in Additional file 1: Text S2 [22]. An overall risk factor burden was calculated and then categorized into three levels: optimal (all risk factors were optimal), borderline (the borderlines were presented but no elevated factors were present), and elevated (any one of the risk factors was elevated) [20].

**Table 1:** Definitions of risk factors

Risk factor and category	Definition
**Smoking**	
**Optimal**	Never smoker
**Borderline**	Former smoker
**Elevated**	Current smoker
**Alcohol consumption**	
**Optimal**	Consumption of < 6 standard drinks/day for women or < 8 standard drinks/day for men
**Elevated**	Consumption of ≥ 6 standard drinks/day for women or ≥ 8 standard drinks/day for men
**Body mass index**	
**Optimal**	< 25
**Borderline**	25–29
**Elevated**	≥ 30
**Blood pressure**	
**Optimal**	Systolic blood pressure < 120 mm Hg and diastolic blood pressure < 80 mm Hg, and no treatment or history for hypertension
**Borderline**	Systolic blood pressure 120-139 mm Hg or diastolic blood pressure 80-89 mm Hg, and no treatment or history for hypertension
**Elevated**	Systolic blood pressure ≥140 mm Hg or diastolic blood pressure ≥ 90 mm Hg, and/or treatment or history for hypertension
**Diabetes mellitus (type 1 or 2)**	
**Optimal**	Glycated hemoglobin (HbA1c) < 6.5 and no treatment or history for diabetes
**Elevated**	Glycated hemoglobin (HbA1c) ≥ 6.5 or treatment or history for diabetes
**History of heart failure or myocardial infarction**	
**Optimal**	No history of heart failure or myocardial infarction
**Elevated**	History of heart failure or myocardial infarction

Furthermore, a risk factor profile was constructed to reflect the risk factor burden with different numbers of borderline/elevated risk factors, which was categorized into 7 groups: “0/0,” “0/1,” “0/2,” “0/≥3,” “1/any,” “2/any,” and “≥3/any.”

### Definition of genetic predisposition

To estimate the genetic predisposition of AF, we constructed a polygenic risk score for each participant, which was derived from the current optimal genetic risk variant list from the meta-analyses excluding the UK Biobank participants (*n*=165 SNPs, Additional file 1: Text S3, and Table S1) [15]. Briefly, most of the genome-wide significant risk variants for AF fall in genes that cause serious heart defects in humans (e.g., PITX2, TBX5) or near genes important for striated muscle function and integrity (e.g., CFL2, MYH7), which are crucial for the function of cardiac ion channels and calcium signaling. According to the number of risk alleles, we used imputed data to calculate the PRS through multiplying by the regression coefficient obtained from the previous study [23]: PRS = (β_1_ × SNP_1_ + β_2_ × SNP_2_ + … + β_165_ × SNP_165_). Furthermore, we classified each participant into three categories: low (lowest quartile), intermediate (mid two quartiles), and high (highest quartile) genetic AF risk groups.

### Statistical analysis

Baseline characteristics including risk factors and PRS were reported as a frequency (proportion) and were compared across different index ages using the chi-square test. To investigate the potential interactions between risk factor burden and PRS on both additive and multiplicative scales within strata of index age and sex, a new term with nine categories representing nine combinations (3×3) of risk factor burden levels and PRS levels was created.

To determine an additive interaction, both the attributable proportion (AP) and relative excess risks due to interaction (RERI), and their 95% confidence intervals (CIs) with the reference group of optimal risk factor burden/profiles and low PRS were calculated, and these measures represented the combined excess risk in both exposed groups [24]. The *P*-values for the additive scale in each index age were adjusted by FDR (false discovery rate). For the multiplicative scale, likelihood tests were performed to compare the model with and without the multiplicative interaction term.

The 10-year risk for incident AF from index ages of 45, 55, and 65 years up to a maximum of 10 years was calculated for each risk factor burden/profile and each risk factor separately, and then we performed subgroup analyses stratified by sex and PRS. To account for the competing death risk and to avoid inflating the cumulative incidence, a modified Kaplan-Meier estimate with age as the time scale was used to compute the 10-year risks of AF and associated 95% CIs [25]. Z ratio tests using low level as the reference were compared to the 10-year risks in the elevated and borderline risk groups.

Multivariable Fine and Gray models for each index age were fitted to predict the 10-year risk of AF (full details in Additional file 1: Text S4) [26, 27]. Calibration plots and C-index were used to evaluate the performance of the models. The predicted 10-year risks of AF for 16 risk profiles were presented in different sexes and PRS separately. All analyses were conducted using SAS software, version 9.4 (SAS Institute), and R software 4.1.1 (R Development Core Team, Vienna, Austria).

## Results

### Characteristics at index ages 45, 55, and 65 years

A total of 348,904 participants were included in statistical analysis defined by index ages of 45 (*n*=84,206), 55 (*n*=117,520), and 65 (*n*=147,178) years (Table 2 and Fig. 1). Among participants at index ages 45, 55, and 65 years, 924 (1.1%), 3883 (3.3%), and 12,924 (8.8%) developed AF during follow-up, respectively. Within each respective group, 45.6%, 43.6%, and 46.9% were men and 90.1%, 94.2%, and 96.9% were white. As index age increased, the proportion of participants that reported smoking decreased, while blood pressure, prevalent diabetes, and history of MI or HF increased. Elevated blood pressure was the most common risk factor (33.8% at age 45 years, 52.5% at 55 years, and 70.9% at 65 years). The PRS of AF ranged from 7.10 to 11.67 with a median of 9.29. Differences in baseline characteristics between low, intermediate, and high PRS at different index ages are presented in Additional file 1: Table S2-4.

**Table 2:** Characteristics of participants

Variables	45 years (***N*** = 84,206)	55 years (***N*** = 117,520)	65 years (***N*** = 147,178)	***p*** value
**Sex**				<.0001
**Female**	45,841 (54.4)	66,283 (56.4)	78,119 (53.1)	
**Male**	38,365 (45.6)	51,237 (43.6)	69,059 (46.9)	
**Ethnicity**				<.0001
**Non-White**	8320 (9.9)	6783 (5.8)	4564 (3.1)	
**White**	75,886 (90.1)	110,737 (94.2)	142,614 (96.9)	
**Smoking**				<.0001
**Optimal**	51,565 (61.2)	66,321 (56.4)	74,110 (50.4)	
**Borderline**	20,966 (24.9)	38,239 (32.5)	61,075 (41.5)	
**Elevated**	11,675 (13.9)	12,960 (11.0)	11,993 (8.1)	
**Alcohol consumption**				<.0001
**Optimal**	81,845 (97.2)	114,010 (97.0)	143,757 (97.7)	
**Elevated**	2361 (2.8)	3510 (3.0)	3421 (2.3)	
**BMI**				<.0001
**Optimal**	32,605 (38.7)	39,883 (33.9)	44,801 (30.4)	
**Borderline**	33,340 (39.6)	48,407 (41.2)	66,694 (45.3)	
**Elevated**	18,261 (21.7)	29,230 (24.9)	35,683 (24.2)	
**Blood pressure**				<.0001
**Optimal**	21,228 (25.2)	16,632 (14.2)	9135 (6.2)	
**Borderline**	34,490 (41.0)	39,243 (33.4)	33,749 (22.9)	
**Elevated**	28,488 (33.8)	61,645 (52.5)	104,294 (70.9)	
**Diabetes mellitus**				<.0001
**Optimal**	81,713 (97.0)	111,303 (94.7)	135,551 (92.1)	
**Elevated**	2493 (3.0)	6217 (5.3)	11,627 (7.9)	
**Heart history**				<.0001
**Optimal**	83,751 (99.5)	115,620 (98.4)	141,774 (96.3)	
**Elevated**	455 (0.5)	1900 (1.6)	5404 (3.7)	
**Polygenic risk score**				<.0001
**Low**	21,118 (25.0)	29,380 (25.0)	37,730 (25.6)	
**Intermediate**	41,910 (49.8)	58,811 (50.1)	74,193 (50.4)	
**High**	21,178 (25.2)	29,329 (25.0)	35,255 (24.0)	

### Single risk factors, risk factor burden, and 10-year risk of atrial fibrillation

The overall 10-year risks of AF were 0.67% (95% CI: 0.61% to 0.73%) for index age 45 years, 2.05% (95% CI: 1.96% to 2.13%) for index age 55 years, and 6.34% (95% CI: 6.21% to 6.46%) for index age 65 years. The 10-year risk of AF increased gradually at each index age with an increase in each single risk factor profile. History of MI or HF had the strongest association with a 10-year risk of AF at each index age, followed by diabetes mellitus and alcohol consumption (Additional file 1: Table S5).

**Fig. 2:**
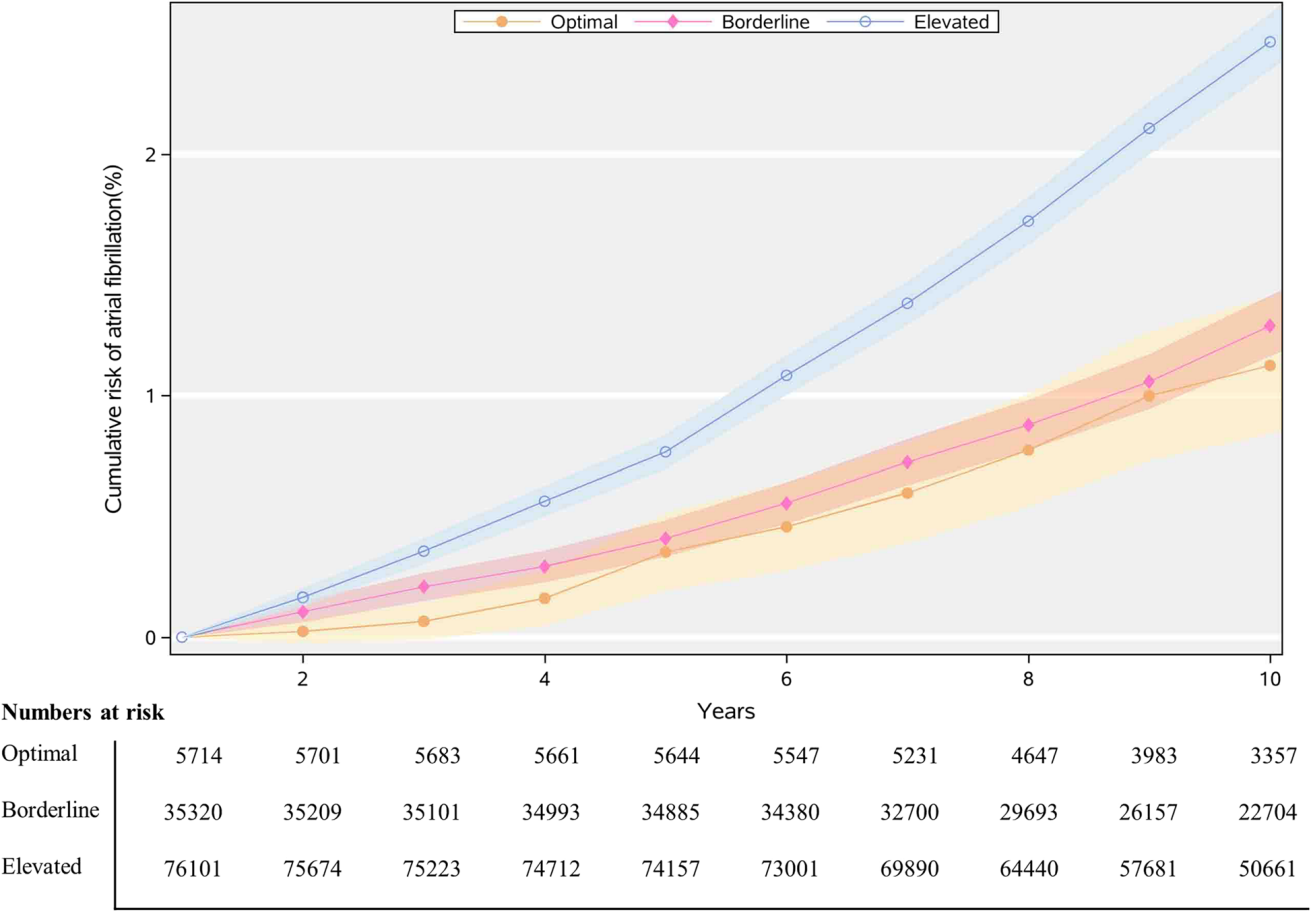
Cumulative risk (%) for atrial fibrillation according to risk factor burdens (optimal, borderline, or elevated) at index age 55 years. Shading = 95% confidence intervals. Participants entered the study sample between ages 55 and < 60 years; therefore, the number at risk increased from age 55 years to < 60 years

Table 3, Fig. 2, and Additional file 2: Figure S1-S2 show the 10-year risks of AF for index ages 45, 55, and 65 years, respectively, with the elevated, borderline, or optimal risk factor burden. The 10-year risk was the lowest for the participants with optimal factors, whereas the risk was higher for participants with elevated factors for index age 45, 55, and 65 years (Table 3). The 10-year AF risks were all higher in men than in women (Additional file 1: Table S6 & S7).

**Table 3:** Ten-year risk (%) of atrial fibrillation in all and different PRS according to risk factor burden, after the adjustment for competing risk of death

Study sample (index age) and risk factor burden	All		Low PRS		Intermediate PRS		High PRS	
	No. of atrial fibrillation cases/total	10-year risk (95% CI)	No. of atrial fibrillation cases/total	10-year risk (95% CI)	No. of atrial fibrillation cases/total	10-year risk (95% CI)	No. of atrial fibrillation cases/total	10-year risk (95% CI)
**45 years**								
**Optimal**	30/7913	0.25 (0.13, 0.36)	5/1974	0.26 (0.03, 0.50)	10/3904	0.18 (0.03, 0.32)	15/2035	0.37 (0.10, 0.64)
**Borderline**	237/32,184	0.44 (0.36, 0.51)^*^	30/8109	0.27 (0.15, 0.38)	94/16,056	0.35 (0.25, 0.44)^*^	113/8019	0.79 (0.59, 0.99)^*^
**Elevated**	657/44,109	0.92 (0.83, 1.01)^*^	90/11,035	0.54 (0.39, 0.68)^*^	283/21,950	0.74 (0.63, 0.86)^*^	284/11,124	1.65 (1.40, 1.90)^*^
**55 years**								
**Optimal**	88/5719	1.13 (0.84, 1.41)	9/1444	0.43 (0.09, 0.78)	41/2828	0.98 (0.60, 1.35)	38/1447	2.10 (1.34, 2.86)
**Borderline**	698/35,405	1.29 (1.17, 1.41)	80/8865	0.61 (0.44, 0.78)	294/17,778	1.02 (0.87, 1.18)	324/8762	2.52 (2.17, 2.86)
**Elevated**	3097/76,396	2.47 (2.35, 2.58)^*^	415/19,071	1.28 (1.11, 1.45)^*^	1421/38,205	2.18 (2.03, 2.33)^*^	1261/19,120	4.22 (3.92, 4.52)^*^
**65 years**								
**Optimal**	111/2867	2.93 (2.29, 3.57)	24/776	2.27 (1.16, 3.38)	35/1380	1.71 (1.00, 2.42)	52/711	6.02 (4.23, 7.81)
**Borderline**	1551/29,686	3.88 (3.65, 4.11)^*^	253/7541	2.43 (2.07, 2.79)	693/15,056	3.44 (3.14, 3.74)^*^	605/7089	6.35 (5.76, 6.94)
**Elevated**	11262/11,4625	7.05 (6.90, 7.20)^*^	1751/29,413	3.91 (3.68, 4.14)^*^	5448/57,757	6.72 (6.51, 6.93)^*^	4063/27,455	11.10 (10.72, 11.49)^*^

*Abbreviations*: *CI* confidence interval, *PRS* polygenic risk score

^*^Test comparing lifetime risk in borderline and elevated risk groups with optimal risk group by z ratio test (that is, the difference in lifetime risk between two groups divided by its standard error). Significant differences between subgroup effects with *p*-values < 0.05

### Potential interactions between risk factor burden and PRS on the AF incidence

Table 4 showed the combined health impact of risk factor burden and PRS on AF incidence. At each index age, compared to those with optimal risk factor burden or low PRS, participants with higher risk factor burden or higher PRS generally had a higher risk of AF. Furthermore, compared with those who had optimal risk factor burden and low PRS, participants with an elevated risk factor burden and high PRS had the highest risk of developing AF at index age 45 years (HR: 7.32; 95% CI: 3.02 to 17.70), 55 years (HR: 8.41; 95% CI: 4.37 to 16.20), and 65 years (HR: 4.12; 95% CI: 2.76 to 6.14).

**Table 4:** Combined effects of risk factor burden and polygenic risk score and atrial fibrillation incidence

Study sample (index age) and risk factor burden	PRS levels (HR, 95% CI)^**a**^			RERI ^**b**^		***P*** for interaction^**c**^
	Low	Intermediate	High	Intermediate	High	
**45 years**						
**Optimal**	1.00	1.01 (0.35, 2.96)	2.89 (1.05, 7.95)^*^			0.63
**Borderline**	1.14 (0.44, 2.93)	1.81 (0.74, 4.46)	4.45 (1.82, 10.88)^*^	0.65 (−0.25, 1.55)	1.15 (−0.55, 2.84)	
**Elevated**	2.28 (0.93, 5.62)	3.65 (1.51, 8.84)^*^	7.32 (3.02, 17.70)^*^	1.43 (0.43, 2.42)^*^	3.35 (0.61, 6.08)^*^	
**55 years**						
**Optimal**	1.00	2.37 (1.15, 4.86)^*^	4.32 (2.09, 8.94)^*^			0.08
**Borderline**	1.24 (0.62, 2.47)	2.28 (1.17, 4.42)^*^	5.17 (2.66, 10.02)^*^	−0.35 (−1.56, 0.85)	0.42 (−1.13, 1.97)	
**Elevated**	2.67 (1.38, 5.16)^*^	4.61 (2.40, 8.89)^*^	8.41 (4.37, 16.20)^*^	0.63 (−0.21, 1.48)	2.44 (0.69, 4.20)^*^	
**65 years**						
**Optimal**	1.00	0.83 (0.50, 1.40)	2.40 (1.49, 3.89)^*^			0.002
**Borderline**	0.97 (0.64, 1.47)	1.33 (0.89, 2.00)	2.56 (1.70, 3.84)^*^	0.53 (0.16, 0.90)^*^	0.18 (−0.59, 0.95)	
**Elevated**	1.54 (1.03, 2.30)^*^	2.51 (1.68, 3.73)^*^	4.12 (2.76, 6.14)^*^	1.13 (0.83, 1.43)^*^	1.16 (0.49, 1.84)^*^	

*Abbreviations*: *PRS* polygenic risk score, *HR* hazard ratio, *CI* confidence interval, *RERI* relative excess risk due to interaction

^a^All results were calculated after adjusting for sex

^b^On the additive scale, the estimates of RERI were calculated based on the reference group with optimal factor burden and low PRS

^c^On the multiplicative scale, likelihood tests were applied to test the significance of the interaction term by comparing the model with and without the interaction term

^*^P-values < 0.05, and the *P*-values for additive scale in each index age were adjusted by FDR (false discovery rate)

On an additive scale, positive interactions were observed for risk factor burden (optimal versus elevated levels) with the PRS at each index age (*P*_additive-scale_ < 0.05, Table 4, Additional file 1: Table S8). For example, for participants at index age 55 with elevated risk factor burden and high PRS, the RERI and AP were 2.44 and 0.29, suggesting that a 2.44 relative excess risk was due to the additive interaction. This accounted for 29% of the risk of AF in participants who had elevated risk factor burden and high PRS. The values of RERIs and APs tended to decrease as index age increased in participants with high PRS and risk factor burden. On a multiplicative scale, significant interactions were found between risk factor burden and PRS in relation to AF risk at index age 65 years (*P*_multiplicative-scale_ < 0.05). Similar results were also observed in the subgroup of sex (Additional file 1: Table S9 and S10).

### Risk factor burden and 10-year risk of AF stratified by genetic predisposition

Among participants without AF at age index age 45, 55, and 65 years, those in optimal risk burdens and low PRS had 10-year risks of AF of 0.26%, 0.43%, and 2.27% separately, whereas those in the elevated risk burdens and high PRS had 10-year risks of AF of 1.65%, 4.22%, and 11.10%, respectively (Table 3).

For individuals at index ages 55 and 65 with optimal risk factor burden and high PRS of AF, the 10-year risks were 2.10% and 6.02%, with values higher than those in other elevated clinical risk factor burden strata at the same index age. At the index age of 45 years, compared with the participants with the optimal burden and high PRS, participants with an elevated risk factor burden and low/intermediate PRS had higher 10-year AF risk. At the same strata of risk factor burden, gradients of increased AF risk were observed with increasing risk of PRS. Stratification analyses by sex were conducted, yielding results that were generally consistent with our main findings (Additional file 1: Table S6 & S7).

### Risk factor profile borderline or multiple elevated risk factors and 10-year risk of AF

Additional file 1: Table S11 shows the distributions of risk factor profiles (number of elevated/borderline risk factors) at age index age 45, 55, and 65 years. The participants with optimal risk factors decreased from 9.4% to 4.9% and 2.0% with the index age changed from 45 to 55 and 65 years, respectively.

As age increased, the number of participants with one or more borderline risk factors decreased, and gradually transitioned to having one to three elevated risk factors. Significant interactions were observed between risk factor profile and PRS in relation to AF risk (Additional file 1: Table S12). Table 5 and Additional file 1: Tables S13 and S14 show the 10-year risk among the participants with elevated or multiple borderline risk factors when the participants were separated into low, intermediate, and high PRS, respectively. The results were similar to the 10-year risks associated with risk factor burden. Especially, the 10-year risks were higher in men with optimal risk factor profiles and high PRS compared to those with elevated risk factor profiles (≥3/any) and low PRS at the index age of 65 years. In contrast, the opposite results were observed in the index age 45 years.

**Table 5:** Ten-year risk (%) of atrial fibrillation by risk factor profiles and number of elevated/borderline risk factors after the adjustment for competing risk of death

Risk factor profile and number of elevated/borderline risk factors	Index age 45 years		Index age 55 years		Index age 65 years	
	No. of atrial fibrillation cases/total	10-year risk (95% CI)	No. of atrial fibrillation cases/total	10-year risk (95% CI)	No. of atrial fibrillation cases/total	10-year risk (95% CI)
**Optimal**						
**0/0**	30/7913	0.25 (0.13, 0.36)	88/5719	1.13 (0.84, 1.41)	111/2867	2.93 (2.29, 3.57)
**Borderline**						
**0/1**	90/15,231	0.36 (0.26, 0.46)	252/14,992	1.08 (0.91, 1.26)	537/11,038	3.63 (3.27, 4.00)
**0/2**	119/13,356	0.48 (0.35, 0.60)	318/15,226	1.37 (1.18, 1.56)	667/12,947	3.81 (3.47, 4.15)
**0/>=3**	28/3597	0.60 (0.34, 0.86)	128/5187	1.65 (1.29, 2.01)	347/5701	4.53 (3.97, 5.08)
**Elevated**						
**1/any**	281/28,166	0.62 (0.52, 0.71)	1302/45,475	1.76 (1.63, 1.88)	5460/69,283	5.55 (5.38, 5.73)
**2/any**	255/12,689	1.27 (1.07, 1.48)	1160/23,820	2.96 (2.73, 3.18)	3997/34,484	8.38 (8.08, 8.69)
**>= 3/any**	121/3254	2.16 (1.64, 2.69)	635/7101	5.36 (4.82, 5.91)	1805/10,858	12.33 (11.69, 12.97)

*Abbreviations*: *CI* confidence interval

### Predicted 10-year risk of AF

**Fig. 3:**
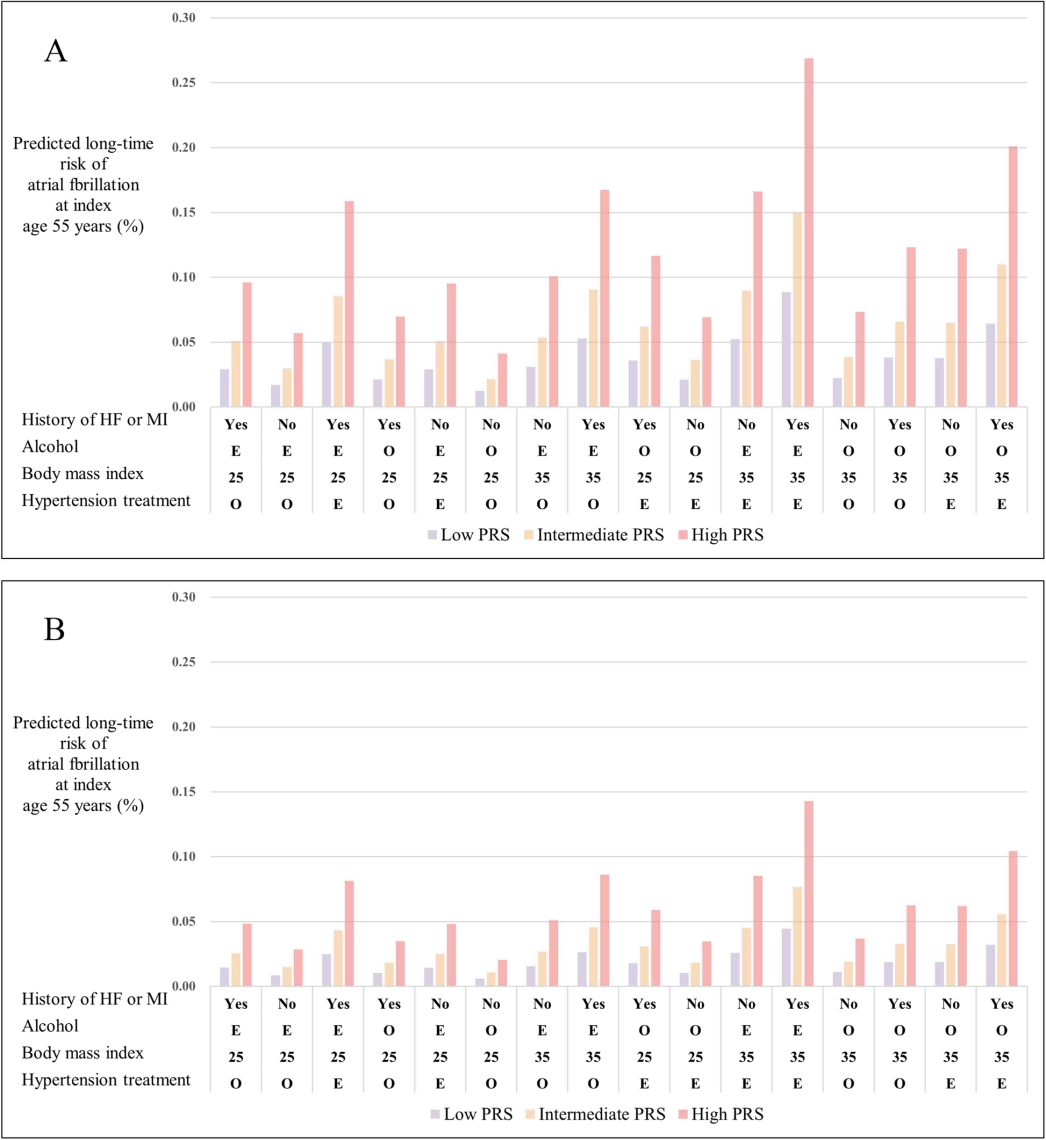
**A** Predicted 10-year risk (%) of atrial fibrillation at index age 55 years across 16 risk profiles, in men with different polygenic risk scores (PRS; low, intermediate, or high). Profiles were defined according to treatment for hypertension (yes or no), body mass index (25 or 35), alcohol (elevated (E) or optimal (O)), and history of myocardial infarction or heart failure (yes or no). For each profile, white participants with a borderline blood pressure level (systolic blood pressure 128 mmHg and diastolic blood pressure 80 mmHg), who never smoked, and who had no diabetes were considered. **B** Predicted 10-year risk (%) of atrial fibrillation at index age 55 years across 16 risk profiles in women with different polygenic risk scores (low, intermediate, or high)

Additional file 1: Table S15-S17 presented three multivariable predictive models of the 10-year risk of AF at index ages 45, 55, and 65 years. The 10-year risk of AF is shown in Fig. 3 and Additional file 2: Figure S3-S6 for 16 different risk profiles for both men and women.

Higher predicted 10-year risks of AF were observed in men and in those with high PRS. Participants with high PRS, history of MI or HF, elevated alcohol consumption, a BMI of 35 or higher, and hypertension treatment had the greatest 10-year risks in both men and women. The C-indexes of the predicted model at index ages 45, 55, and 65 years were 73.5% (95% CI: 71.7% to 75.2%), 71.0% (95% CI: 70.1% to 71.9%), and 67.1% (95% CI: 66.2% to 68.1%), respectively. Additional file 2: Figures S7-S9 show the calibration plots of the predictive models at index ages 45, 55, and 65 years.

## Discussion

To the best of our knowledge, this is the largest prospective cohort study to illustrate the 10-year risk of AF which takes into account both risk factor burden and genetic predisposition. Thus, overall findings were consistent with our study hypothesis, more specifically, among participants aged 45, 55, and 65 years, the overall 10-year risks of AF were 0.67%, 2.05%, and 6.34%, respectively. The 10-year risk of AF at index ages 45, 55, and 65 years ranged from 0.26%, 0.43%, and 2.27% among participants in the lowest tertiles of risk factor burden and genetic predisposition, rising to almost 1.65%, 4.22%, and 11.10% among those in the highest tertiles of risk factor burden and genetic predisposition. The 10-year risk was highest in men with high PRS and elevated risk factor burden at each index age.

There are three key clinical implications to be drawn from this analysis. *First*, risk factor burden/profiles, genetic predisposition, and their interactions play pivotal roles in the risk of AF, especially among male participants and those of a younger age. Especially, the risk of AF attributable to a risk factor burden/profile increased along with the increased risk due to genetic predisposition. *Second*, age was the most prominent risk factor for AF. Along with aging, the contributions of risk factors and genetic predisposition on AF risk decreased, especially among men. *Third*, at younger ages, elevated risk burden/profiles with low/intermediate PRS might lead to earlier onset of AF, compared to the joint effect of high PRS and optimal risk burden/profiles. Hence, an optimal risk burden is important for AF prevention, especially for individuals who are younger or with high genetic predisposition of AF.

### Comparison with other studies

The Framingham Heart Study estimated short-term and lifetime AF risk among 9764 participants without AF at ages 55 to 75 years [21]. Though instrumental in value, cohort studies are needed to further refine the joint effect of both polygenic risk and other risk factors. Our updated 10-year risks, estimated by the contribution of both risk factors burden and genetic predisposition, developed from a substantially larger prospective cohort study, offers superior generalizability to contemporary populations and helps identify high-risk populations [10].

While previous literature has reported that the 10-year risk of AF is 2.0%, 4.5%, and 7.6% among participants in the index age of 55 years with low, intermediate, and high polygenic risk [21], the observed lower 10-year risk in our study may be attributable to a relatively healthier cohort in UK Biobank. Furthermore, the incidence of AF increases rapidly after the age of 40, and the 10-year AF risk varies with age [7].

Previous studies have largely focused on middle-aged and older adults (aged ≥ 55 years old) to investigate the 10-year risk of AF, and it is uncertain of the applicability to younger individuals (age ≤ 55). Indeed, prospectively identifying these relatively young individuals at risk for AF might be beneficial, allowing for early intervention measures [28]. Our study included younger participants (index age 45 years) and observed that the joint effect of elevated risk factors burden/profiles and high inherited predisposition on AF incidence declined with advancing age. Furthermore, among the younger age group, compared to genetic predisposition, risk factor burden/profiles have a larger effect on AF development. Our results suggest that a more favorable risk burden/profile might delay the onset of AF risk, especially among young people and men. Similar to a previous study [21], participants with high polygenic risk had twice the 10-year risk than those with low PRS, underscoring the independent impact of an inherited predisposition to AF risk.

Three AF multivariable predictive models had moderate discrimination (C-index 67.1% to 73.5%) but suboptimal calibration. The performances of our models were comparable to other models reported in prior studies [20, 29]. In our predictive models, the strongest risk factors were sex, history of MI or HF, and PRS. Both the 10-year and predicted risks of AF were higher in men than women, as previously noted [30]. One possible explanation might be that women had more favorable risk burdens compared to men. Furthermore, considering the main outcomes in the present study, which include both AF and flutter, risk factor burden and genetic predisposition might have an independent prognostic impact in terms of different types of AF [31].

Compared to the performances of previously used models, we included ethnicity as an important covariate in the AF prediction models due to the accumulated evidence that white individuals have a higher AF risk than those who are not white [32]. Furthermore, the preponderance of AF cases in our population (56%, 71%, and 82% in the index age of 45, 55, and 65 years, respectively) developed in participants with a history of MI, HF, hypertension, or diabetes, suggesting that comorbidities contributed more to AF risk in older individuals. Additionally, lifestyle risk factors including BMI, alcohol consumption, and smoking, which are modifiable, also played important roles in 10-year risk and our predictive models. Alcohol consumption led to the highest 10-year risk of AF among lifestyle risk factors. There is growing evidence on the adverse effects of alcohol on left atrial function, electrical remolding, and structure. These effects would contribute to AF [33]. Considering the close association of risk factors with the risk of AF, these modifiable factors may be better targets for AF prevention [34].

Furthermore, we hypothesize that our prediction models could be used to further inform targeted screening of individuals at risk, which may be more cost-effective than routinely screening all patients aged ≥65 years [35, 36]. The risk factor burden of AF is an easily interpretable and accessible tool of structured management, which can be readily measured with existing and future data. Moreover, with the increasing ubiquity of genomic data, both in the clinical setting and via direct-to-consumer testing, the current optimal variant list of a PRS for AF could potentially provide a more accurate personalized risk assessment [15, 21]. For individuals at an index age of 45, 55, and 65 with elevated risk factor burden and high PRS, the 10-year risks of AF were 1.65%, 4.22%, and 11.10%, respectively, which could potentially be used as thresholds. The application of the prediction models could be used to inform thresholds for screening in individuals with high predicted 10-year risks, potentially leading to earlier detection, guidance on preventive approaches, and individualized counseling.

### Strengths and limitations

The strengths of this study include the uniquely large sample size, including younger participants; long-term follow-up; detailed information on behavioral, clinical profile, and genetic data; the large number of AF cases included in the analysis; and the estimation of 10-year risks incorporating both risk burden/profiles and genetic predisposition with adjustment for competing risks. Prediction models and potential thresholds were developed in our study to help implement early and targeted interventions.

Our study also has some limitations. Importantly, biomarkers, electrocardiographic data, anemia, electrolyte imbalance, and other potential risk factors for AF were not included in our study [37]. Nonetheless, some studies have pointed out that electrocardiogram data may not improve the performance of predictive models [8, 38]. Some AF events caused by acute events, such as trauma, surgery, etc., may have been missed since the majority of events were ascertained using hospital inpatient data, thus the true risk of AF might be underestimated since undiagnosed AF is common [3]. In addition, measurements of risk factor burden/profiles were attained at baseline and could not reflect changes over time. Participants recruited in the UK Biobank are healthier and with a higher socioeconomic status than the general UK population, which might result in healthy volunteer bias [39]. Given that an adequate number of participants with various levels of exposure were evaluated with high internal validity, this might not have an impact on the valid estimates of relationships [39]. The UK Biobank does not record close relationships or not release the kinship coefficients of all participants estimated from genetic data, which prevents us from implementing mixed-effects models that account for the relatedness among the samples. Finally, our study used only one cohort to predict 10-year risks and predictive models of AF, and whether our findings can be generalized to other ethnic groups needs further investigation.

## Conclusions

An optimal risk factor burden, including maintaining a normal body weight, no smoking, moderate to no alcohol consumption, and treatment for hypertension and diabetes mellitus, may be important for AF prevention, especially among men, participants with high genetic predisposition, or those at a young age. Our predictive model would be helpful to identify high-risk populations for primary AF prevention and facilitate preventive measures.

## Supplementary Information

**Additional file 1: Text S1.** The detail information on quality-controlled genotyping data. **Text S2.** Details of the assessment of covariates. **Text S3.** Detailed definition of genetic predisposition. **Text S4.** Details of multivariable Fine and Gray models and C-index. **Table S1.** Individual atrial fibrillation SNP association atrial fibrillation odds. **Table S2.** Characteristics of participants at the index age of 45 years according to PRS, divided into Low, Intermediate, and High. **Table S3.** Characteristics of participants at the index age of 55 years according to PRS, divided into Low, Intermediate, and High. **Table S4.** Characteristics of participants at the index age of 65 years according to PRS, divided into Low, Intermediate, and High. **Table S5.** 10-year risk (%) of atrial fibrillation by individual risk factors, after adjustment for competing risk of death. **Table S6.** 10-year risk (%) of atrial fibrillation in PRS in men according to the risk factor burden, after adjustment for competing risk of death. **Table S7.** 10-year risk (%) of atrial fibrillation in PRS in women according to the risk factor burden, after adjustment for competing risk of death. **Table S8.** Attributable proportion of risk factor burdens and PRS and AF incidence in overall and by sex. **Table S9.** Combined effects of risk factor burdens and PRS and AF incidence in men. **Table S10.** Combined effects of risk factor burdens and PRS and AF incidence in women. **Table S11.** Distribution of risk factor profiles. **Table S12.** Additive and multiplicative interactions between risk factor profiles and PRS in relation to AF incidence. **Table S13.** 10-year risk (%) of atrial fibrillation in men by risk factor profiles (number of elevated/borderline risk factors) and PRS, after adjustment for competing risk of death. **Table S14.** 10-year risk (%) of atrial fibrillation in women by risk factor profiles (number of elevated/borderline risk factors) and PRS, after adjustment for competing risk of death. **Table S15.** Multivariable prediction model of the 10-year risk of atrial fibrillation at index age 45 years. **Table S16.** Multivariable prediction model of the 10-year risk of atrial fibrillation at index age 55 years. **Table S17.** Multivariable prediction model of the 10-year risk of atrial fibrillation at index age 65 years.

**Additional file 2:** Central illustration. **Figure S1.** Cumulative risk (%) for atrial fibrillation according to risk factor burdens (optimal, borderline, or elevated) at the index age of 45 years. **Figure S2.** Cumulative risk (%) for atrial fibrillation according to risk factor burdens (optimal, borderline, or elevated) at the index age of 65 years. **Figure S3.** Predicted 10-year risk (%) of atrial fibrillation at index age 45 years across 16 risk profiles, in men with different polygenic risk score (low, intermediate, or high). **Figure S4.** Predicted 10-year risk (%) of atrial fibrillation at index age 45 years across 16 risk profiles, in women with different polygenic risk score (low, intermediate, or high). **Figure S5.** Predicted 10-year risk (%) of atrial fibrillation at index age of 65 years across 16 risk profiles, in men with different polygenic risk score (low, intermediate, or high). **Figure S6.** Predicted 10-year risk (%) of atrial fibrillation at index age of 65 years across 16 risk profiles, in women with different polygenic risk score (low, intermediate, or high). **Figure S7.** Calibration plot of the prediction model for predicting 10-year risk at index age of 45 years. **Figure S8.** Calibration plot of the prediction model for predicting 10-year risk at index age 55 years. **Figure S9.** Calibration plot of the prediction model for predicting 10-year risk at index age 65 years.

## Data Availability

Data are available upon reasonable request to the UK Biobank management team (https://www.ukbiobank.ac.uk/media/px5gbq4q/access_019-access-management-system-user-guide-v4-1.pdf ).

## Abbreviations

AF: Atrial fibrillation
PRS: Polygenic risk score
BMI: Body mass index
MI: Myocardial infarction
HF: Heart failure
RERI: Relative excess risks due to interaction
AP: Attributable proportion
CIs: Confidence intervals
FHS: Framingham Heart Study
