# Supplementary material for: Associations of risk factor burden and genetic predisposition with the 10-year risk of atrial fibrillation: observations from a large prospective study of 348,904 participants
Source: BMC Med. 2023 Mar 8;21:88. doi: 10.1186/s12916-023-02798-7 (PMC9993634; doi:10.1186/s12916-023-02798-7)
Supplement: Supplementary file 1 — Additional file 1: Text S1. The detail information on quality-controlled genotyping data. Text S2. Details of the assessment of covariates. Text S3. Detailed definition of genetic predisposition. Text S4. Details of multivariable Fine and Gray models and C-index. Table S1. Individual atrial fibrillation SNP association atrial fibrillation odds. Table S2. Characteristics of participants at the index age of 45 years according to PRS, divided into Low, Intermediate, and High. Table S3. Characteristics of participants at the index age of 55 years according to PRS, divided into Low, Intermediate, and High. Table S4. Characteristics of participants at the index age of 65 years according to PRS, divided into Low, Intermediate, and High. Table S5. 10-year risk (%) of atrial fibrillation by individual risk factors, after adjustment for competing risk of death. Table S6. 10-year risk (%) of atrial fibrillation in PRS in men according to the risk factor burden, after adjustment for competing risk of death. Table S7. 10-year risk (%) of atrial fibrillation in PRS in women according to the risk factor burden, after adjustment for competing risk of death. Table S8. Attributable proportion of risk factor burdens and PRS and AF incidence in overall and by sex. Table S9. Combined effects of risk factor burdens and PRS and AF incidence in men. Table S10. Combined effects of risk factor burdens and PRS and AF incidence in women. Table S11. Distribution of risk factor profiles. Table S12. Additive and multiplicative interactions between risk factor profiles and PRS in relation to AF incidence. Table S13. 10-year risk (%) of atrial fibrillation in men by risk factor profiles (number of elevated/borderline risk factors) and PRS, after adjustment for competing risk of death. Table S14. 10-year risk (%) of atrial fibrillation in women by risk factor profiles (number of elevated/borderline risk factors) and PRS, after adjustment for competing risk of death. Table S15. Multivariable predicti [file 12916_2023_2798_MOESM1_ESM.docx]

**Additional File 1**

**Associations of risk factor burden and genetic predisposition with the 10-year risk of atrial fibrillation: Observations from a large prospective study of 348,904 participants**

Junguo Zhang PhD, Ge Chen PhD, Xiaojie Wang PhD, Zhengmin (Min) Qian PhD, Miao Cai PhD, Michael G. Vaughn PhD, Elizabeth Bingheim PhD, Haitao Li PhD, Yanhui Gao PhD, Gregory Y. H. Lip^+^ MD, Hualiang Lin^+*^ PhD

[^+^Joint senior authors]

*Corresponding author

| Contents | | |
| --- | --- | --- |
| Text S1 | The detail information on quality-controlled genotyping data | Page 4 |
| Text S2 | Details of the assessment of covariates | Page 4 |
| Text S3 | Detailed definition of genetic predisposition | Page 4 |
| Text S4 | Details of multivariable Fine and Gray models and C-index | Page 4-5 |
| Table S1 | Individual atrial fibrillation SNP association atrial fibrillation odds | Page 6-9 |
| Table S2 | Characteristics of participants at the index age of 45 years according to PRS, divided into Low, Intermediate, and High | Page 10 |
| Table S3 | Characteristics of participants at the index age of 55 years according to PRS, divided into Low, Intermediate, and High | Page 11 |
| Table S4 | Characteristics of participants at the index age of 65 years according to PRS, divided into Low, Intermediate, and High | Page 12 |
| Table S5 | 10-year risk (%) of atrial fibrillation by individual risk factors, after adjustment for competing risk of death | Page 13-14 |
| Table S6 | 10-year risk (%) of atrial fibrillation in PRS in men according to the risk factor burden, after adjustment for competing risk of death | Page 14 |
| Table S7 | 10-year risk (%) of atrial fibrillation in PRS in women according to the risk factor burden, after adjustment for competing risk of death | Page 15 |
| Table S8 | Attributable proportion of risk factor burdens and PRS and AF incidence in overall and by sex | Page 16 |
| Table S9 | Combined effects of risk factor burdens and PRS and AF incidence in men | Page 17 |
| Table S10 | Combined effects of risk factor burdens and PRS and AF incidence in women | Page 18 |
| Table S11 | Distribution of risk factor profiles | Page 19 |
| Table S12 | Additive and multiplicative interactions between risk factor profiles and PRS in relation to AF incidence | Page 20 |
| Table S13 | 10-year risk (%) of atrial fibrillation in men by risk factor profiles (number of elevated/borderline risk factors) and PRS, after adjustment for competing risk of death | Page 21-22 |
| Table S14 | 10-year risk (%) of atrial fibrillation in women by risk factor profiles (number of elevated/borderline risk factors) and PRS, after adjustment for competing risk of death | Page 23-24 |
| Table S15 | Multivariable prediction model of the 10-year risk of atrial fibrillation at index age 45 years | Page 25 |
| Table S16 | Multivariable prediction model of the 10-year risk of atrial fibrillation at index age 55 years | Page 26 |
| Table S17 | Multivariable prediction model of the 10-year risk of atrial fibrillation at index age 65 years | Page 27 |

**Text S1. The detail information on quality-controlled genotyping data**

To minimize the potential confounding caused by ancestry, our main analysis only included unrelated participants. We excluded participants (n = 95,175) according to UK Biobank Data Field 22020, In detail, we applied individual-level quality control (QC) to exclude individuals who were outliers for heterozygosity or missing rates, with a missing rate >0.02 on autosomes (n=968), with sex discordance (between the phenotypic and genetically inferred sex) (n=372), individuals with sex chromosome aneuploidy (n=651), and who were not in a maximal set of unrelated individuals up to 3rd degree (n=93,184), leaving 405, 988 unrelated participants.

**Text S2. Details of the assessment of covariates**

Smoking status was classified into never smoker, former smoker, and current smoker.

Elevated alcohol consumption was defined as ≥6 standard drinks /day for women or ≥8 standard drinks/day for men. Detailed information on alcohol consumption and binge drinking in the UK Biobank has been reported previously [21].

BMI was calculated using height and weight measured at the baseline.

Information on history of disease [hypertension (ICD code I10/I15), diabetes mellitus (ICD code E10-E14), myocardial infarction (ICD code I21-I23, I24.1, I25.2)] and heart failure (ICD code I50, I110)] was extracted from its first occurrence. This information was gathered from self-reported outcomes, primary care, hospital admissions, and death records, and were converted into three-digit ICD-10 categories. We also collected cases directly from hospital admissions and self-reported outcomes.

Information on antihypertensives and antidiabetics were collected by asking “Do you regularly take any of the following medications? (You can select more than one answer)” and participants could select the answer from a list of medications which included blood pressure medication and insulin. Additionally, medication use including antihypertensives and antidiabetics were extracted through treatment/medication records.

Furthermore, we also considered ethnicity (white and non-white) as an important covariate in the AF prediction models.

**Text S3: Detailed definition of genetic predisposition**

The UK Biobank imputed data was used in the current study. The UK Biobank carried out imputation on the genotype data using SHAPEIT3 and IMPUTE4 to statistically infer the genotypes of variants that had not been directly called in the genotyping array, and those which were missing or had been set to missing in central UKB quality control.

Based on the variant list reported by Nielsen et al. [15], 165 variants were available in the UKB imputed genetic data, and there were no multi-allelic or ambiguous SNPs.

**Text S4. Details of multivariable Fine and Gray models and C-index**

We developed three prediction models for the 10-year risk of atrial fibrillation (AF) at the index ages of 45, 55, and 65 years, respectively. We used three multivariable Fine-Gray models accounting for the competing risk of death. We included 11 covariates: Body mass index (BMI), systolic blood pressure, and diastolic blood pressure as continuous variables, and sex, smoking status (never, past, or current), alcohol intake (optimal or elevated), diabetes (optimal, borderline, or elevated), treatment or history for hypertension, history of myocardial infarction or heart failure as categorical variables, and polygenic risk score (PRS; low, intermediate, or high). We did not perform any variable selection. We checked the assumptions of the Fine and Gray model. We found that the proportional hazards assumptions were violated for treatment or history for hypertension in index age 45 model; sex, and SBP in index age 55 model; and sex, SBP, DBP, treatment or history for hypertension and PRS in index age 65 model. As a consequence, we added time-varying covariates Z × log(t) to the model.

To estimate C-index, 10 of the bootstrap sets were used for cross-validation [25,26]. Specially, a bootstrap subsampling version of the .632+ estimate was used, where the size of the training sets is set at 63.2% times the full sample size. Also, the C-index was averaged across the 10 splits.

**Table S1 Individual atrial fibrillation SNP association atrial fibrillation odds**

| **Rs ID** | **Position** | **Risk/reference allele** | **Effect (beta)** | **StdErr** | **P-value** |
| --- | --- | --- | --- | --- | --- |
| rs284277 | chr1:10790797 | C/A | 0.0422 | 0.0069 | 1.245e-09 |
| rs7529220 | chr1:22282619 | C/T | 0.0621 | 0.0098 | 1.983e-10 |
| rs2885697 | chr1:41544279 | G/T | 0.0439 | 0.007 | 2.884e-10 |
| rs11590635 | chr1:49309764 | A/G | 0.1456 | 0.0248 | 4.123e-09 |
| rs146518726 | chr1:51535039 | A/G | 0.1605 | 0.0207 | 8.27e-15 |
| rs1545300 | chr1:112464004 | C/T | 0.0558 | 0.0073 | 1.481e-14 |
| rs4073778 | chr1:116297758 | A/C | 0.0486 | 0.0067 | 4.956e-13 |
| rs10465885 | chr1:147232740 | C/T | 0.0302 | 0.0067 | 5.735e-06 |
| rs79187193 | chr1:147255831 | G/A | 0.1162 | 0.0153 | 3.15e-14 |
| rs6689306 | chr1:154395946 | A/G | 0.046 | 0.0068 | 1.36e-11 |
| rs4999127 | chr1:154714006 | A/G | 0.0827 | 0.0098 | 4.279e-17 |
| rs11264280 | chr1:154862952 | T/C | 0.1347 | 0.0071 | 3.067e-79 |
| rs72700114 | chr1:170193825 | C/G | 0.2021 | 0.013 | 3.288e-54 |
| rs72700118 | chr1:170194823 | A/C | 0.1227 | 0.0101 | 9.524e-34 |
| rs577676 | chr1:170587340 | C/T | 0.0923 | 0.0067 | 1.62e-43 |
| rs10753933 | chr1:203026214 | T/G | 0.0609 | 0.0067 | 9.844e-20 |
| rs4951258 | chr1:205691316 | A/G | 0.0376 | 0.0067 | 2.104e-08 |
| rs7578393 | chr2:26165528 | T/C | 0.0614 | 0.0088 | 2.42e-12 |
| rs11689011 | chr2:46541176 | T/C | 0.0321 | 0.0103 | 3.124e-08 |
| rs11125871 | chr2:61470126 | C/T | 0.0394 | 0.0068 | 6.416e-09 |
| rs2540949 | chr2:65284231 | A/T | 0.0659 | 0.0068 | 2.949e-22 |
| rs6747542 | chr2:70106832 | T/C | 0.0554 | 0.0067 | 1.096e-16 |
| rs72926475 | chr2:86594487 | G/A | 0.0683 | 0.0102 | 2.372e-11 |
| rs28387148 | chr2:127433465 | T/C | 0.0741 | 0.0113 | 6.251e-11 |
| rs67969609 | chr2:145760353 | G/C | 0.0711 | 0.0126 | 1.712e-08 |
| rs56181519 | chr2:175555714 | C/T | 0.0662 | 0.0077 | 6.463e-18 |
| rs2288327 | chr2:179411665 | G/A | 0.0919 | 0.0089 | 7.257e-25 |
| rs3820888 | chr2:201180023 | C/T | 0.0684 | 0.0068 | 5.748e-24 |
| rs35544454 | chr2:213266003 | A/T | 0.0589 | 0.0087 | 1.1e-11 |
| rs7650482 | chr3:12841804 | G/A | 0.0711 | 0.007 | 1.793e-24 |
| rs73041705 | chr3:24463235 | T/C | 0.0443 | 0.0073 | 1.546e-09 |
| rs7374540 | chr3:38634142 | A/C | 0.0325 | 0.0068 | 1.682e-06 |
| rs7373065 | chr3:38710315 | T/C | 0.2024 | 0.0251 | 7.584e-16 |
| rs6790396 | chr3:38771925 | G/C | 0.0627 | 0.0068 | 2.401e-20 |
| rs34080181 | chr3:66454191 | G/A | 0.0446 | 0.0069 | 1.278e-10 |
| rs17005647 | chr3:69406181 | T/C | 0.0413 | 0.0069 | 2.698e-09 |
| rs10804493 | chr3:111554426 | A/G | 0.0558 | 0.007 | 1.629e-15 |
| rs1278493 | chr3:135814009 | G/A | 0.0389 | 0.0068 | 8.769e-09 |
| rs13077048 | chr3:141106954 | T/A | 0.0493 | 0.0104 | 4.75e-10 |
| rs62274627 | chr3:148702947 | A/G | 0.0297 | 0.0109 | 2.458e-08 |
| rs7612445 | chr3:179172979 | T/G | 0.0493 | 0.0084 | 4.808e-09 |
| rs60902112 | chr3:194800853 | T/C | 0.0445 | 0.0079 | 1.719e-08 |
| rs34104130 | chr4:10101300 | G/T | 0.0365 | 0.0112 | 2.552e-11 |
| rs1458038 | chr4:81164723 | T/C | 0.0434 | 0.0072 | 1.735e-09 |
| rs6841049 | chr4:83910712 | T/G | 0.0242 | 0.0103 | 1.954e-08 |
| rs10006327 | chr4:103890980 | C/T | 0.0364 | 0.0067 | 4.423e-08 |
| rs244017 | chr4:111255917 | T/G | 0.0194 | 0.0085 | 0.02247 |
| rs61501369 | chr4:111524629 | T/C | 0.1038 | 0.008 | 4.686e-38 |
| rs6850025 | chr4:111596360 | A/G | 0.1815 | 0.0154 | 4.93e-32 |
| rs67249485 | chr4:111699685 | T/A | 0.3655 | 0.0081 | 7.32e-443 |
| rs3853445 | chr4:111761487 | T/C | 0.1693 | 0.0076 | 3.60e-109 |
| rs79399769 | chr4:111925656 | C/T | 0.1197 | 0.0232 | 2.591e-07 |
| rs1532170 | chr4:112165212 | G/A | 0.0306 | 0.007 | 1.258e-05 |
| rs138311480 | chr4:112454295 | C/T | 0.0739 | 0.0284 | 0.00928 |
| rs114904067 | chr4:112604821 | G/A | 0.082 | 0.0215 | 0.0001364 |
| rs7687819 | chr4:113329345 | A/G | 0.0218 | 0.0079 | 0.00612 |
| rs6829664 | chr4:114448656 | G/A | 0.0556 | 0.0076 | 1.922e-13 |
| rs10213171 | chr4:148937537 | G/C | 0.091 | 0.0134 | 1.323e-11 |
| rs10520260 | chr4:174447349 | A/G | 0.0457 | 0.0073 | 3.362e-10 |
| rs12648245 | chr4:174641184 | T/C | 0.0926 | 0.0127 | 3.454e-13 |
| rs6596717 | chr5:106427609 | C/A | 0.0404 | 0.0068 | 3e-09 |
| rs337705 | chr5:113737062 | G/T | 0.0564 | 0.0068 | 1.634e-16 |
| rs2012809 | chr5:128190363 | G/A | 0.0582 | 0.0094 | 4.923e-10 |
| rs2040862 | chr5:137419989 | T/C | 0.1084 | 0.0087 | 1.077e-35 |
| rs17118812 | chr5:139703286 | C/T | 0.0431 | 0.0114 | 1.827e-10 |
| rs6580277 | chr5:142818123 | G/A | 0.067 | 0.0079 | 1.643e-17 |
| rs12188351 | chr5:168386089 | A/G | 0.0865 | 0.0145 | 2.516e-09 |
| rs6891790 | chr5:172670745 | G/T | 0.0729 | 0.0076 | 4.531e-22 |
| rs28439930 | chr5:173393111 | G/C | 0.0458 | 0.0068 | 1.186e-11 |
| rs73366713 | chr6:16415751 | G/A | 0.1035 | 0.0099 | 1.531e-25 |
| rs34969716 | chr6:18210109 | A/G | 0.0702 | 0.0078 | 1.595e-19 |
| rs2308655 | chr6:31322303 | C/G | 0.0494 | 0.012 | 8.085e-09 |
| rs3176326 | chr6:36647289 | G/A | 0.0626 | 0.0085 | 1.424e-13 |
| rs12211255 | chr6:76188330 | A/C | 0.057 | 0.0159 | 2.596e-10 |
| rs2031522 | chr6:87821501 | A/G | 0.0436 | 0.0068 | 1.465e-10 |
| rs3951016 | chr6:118559658 | A/T | 0.0648 | 0.0067 | 2.149e-22 |
| rs9401451 | chr6:122099152 | G/A | 0.0733 | 0.011 | 2.513e-11 |
| rs13195459 | chr6:122403559 | G/A | 0.0623 | 0.007 | 4.152e-19 |
| rs4896104 | chr6:135119089 | C/T | 0.0421 | 0.0103 | 8.615e-10 |
| rs117984853 | chr6:149399100 | T/G | 0.1228 | 0.012 | 1.342e-24 |
| rs12700233 | chr7:904757 | T/G | 0.0367 | 0.0104 | 5.204e-11 |
| rs55734480 | chr7:14372009 | A/G | 0.0548 | 0.0078 | 2.201e-12 |
| rs6462079 | chr7:28415827 | A/G | 0.0466 | 0.0076 | 8.793e-10 |
| rs35005436 | chr7:74134911 | C/T | 0.0612 | 0.0097 | 3.342e-10 |
| rs56201652 | chr7:92278116 | G/A | 0.0531 | 0.0075 | 1.743e-12 |
| rs2283038 | chr7:106835410 | T/C | 0.0266 | 0.012 | 9.756e-10 |
| rs11773845 | chr7:116191301 | A/C | 0.1054 | 0.0067 | 2.39e-55 |
| rs55985730 | chr7:128417044 | G/T | 0.0867 | 0.0149 | 5.236e-09 |
| rs7789146 | chr7:150661409 | G/A | 0.0584 | 0.0087 | 2.116e-11 |
| rs35620480 | chr8:11499908 | C/A | 0.054 | 0.0092 | 5.152e-09 |
| rs7508 | chr8:17913970 | A/G | 0.0711 | 0.0075 | 1.691e-21 |
| rs7834729 | chr8:21821778 | G/T | 0.0653 | 0.0104 | 3.553e-10 |
| rs17430364 | chr8:118863445 | T/A | 0.0425 | 0.0133 | 4.433e-08 |
| rs62521286 | chr8:124551975 | G/A | 0.1202 | 0.0135 | 4.498e-19 |
| rs4871397 | chr8:124635197 | G/C | 0.0756 | 0.0138 | 4.646e-08 |
| rs35006907 | chr8:125859817 | A/C | 0.0083 | 0.0107 | 2.663e-08 |
| rs72721963 | chr8:135798224 | G/A | 0.039 | 0.0174 | 1.907e-09 |
| rs6994744 | chr8:141740868 | C/A | 0.0405 | 0.0066 | 1.095e-09 |
| rs10821415 | chr9:97713459 | A/C | 0.0821 | 0.0067 | 2.924e-34 |
| rs4743034 | chr9:109632353 | A/G | 0.0246 | 0.0122 | 6.139e-09 |
| rs10760361 | chr9:127178266 | G/T | 0.0259 | 0.0106 | 6.035e-10 |
| rs2274115 | chr9:139094773 | G/A | 0.0487 | 0.0076 | 1.691e-10 |
| rs12245149 | chr10:65321147 | C/A | 0.047 | 0.0067 | 1.664e-12 |
| rs7096385 | chr10:69664881 | T/C | 0.0707 | 0.013 | 4.871e-08 |
| rs60212594 | chr10:75414344 | G/C | 0.1176 | 0.0096 | 9.199e-35 |
| rs10458660 | chr10:77936576 | G/A | 0.0537 | 0.0087 | 6.783e-10 |
| rs55693294 | chr10:105277474 | T/C | 0.0546 | 0.0146 | 0.0001819 |
| rs11598047 | chr10:105342672 | G/A | 0.1537 | 0.009 | 8.952e-66 |
| rs35176054 | chr10:105480387 | A/T | 0.1391 | 0.01 | 3.207e-44 |
| rs10749053 | chr10:112576695 | T/C | 0.0555 | 0.0097 | 1.049e-08 |
| rs10741807 | chr11:20011445 | T/C | 0.0729 | 0.0079 | 1.591e-20 |
| rs565449 | chr11:95092398 | G/A | 0.0409 | 0.0109 | 2.235e-09 |
| rs4935786 | chr11:121661507 | T/A | 0.0463 | 0.0079 | 4.854e-09 |
| rs76097649 | chr11:128764570 | A/G | 0.1151 | 0.0124 | 1.26e-20 |
| rs2291437 | chr12:24715048 | G/T | 0.0955 | 0.0104 | 5.052e-20 |
| rs4963776 | chr12:24779491 | G/T | 0.0913 | 0.0088 | 1.839e-25 |
| rs17380837 | chr12:26345526 | C/T | 0.0501 | 0.0072 | 4.799e-12 |
| rs12809354 | chr12:32978437 | C/T | 0.0718 | 0.0094 | 2.886e-14 |
| rs11614818 | chr12:56055815 | C/T | 0.0329 | 0.007 | 2.441e-06 |
| rs2860482 | chr12:57105938 | A/C | 0.054 | 0.0076 | 1.212e-12 |
| rs71454237 | chr12:70013415 | G/A | 0.062 | 0.0084 | 1.784e-13 |
| rs775498 | chr12:70071513 | G/A | 0.0423 | 0.0074 | 1.053e-08 |
| rs12426679 | chr12:76237987 | C/T | 0.0391 | 0.0067 | 4.945e-09 |
| rs883079 | chr12:114793240 | T/C | 0.0981 | 0.0074 | 2.837e-40 |
| rs116904997 | chr12:120668534 | G/A | 0.1116 | 0.0405 | 1.275e-08 |
| rs10773657 | chr12:123327900 | C/A | 0.0575 | 0.0103 | 2.537e-08 |
| rs7134121 | chr12:124447346 | T/C | 0.0317 | 0.0108 | 2.003e-11 |
| rs6560886 | chr12:133150210 | C/T | 0.051 | 0.009 | 1.491e-08 |
| rs9506925 | chr13:23368943 | T/C | 0.0449 | 0.0075 | 2.716e-09 |
| rs1980728 | chr13:47247985 | G/T | 0.0361 | 0.0116 | 2.083e-09 |
| rs35569628 | chr13:113872712 | T/C | 0.0452 | 0.008 | 1.378e-08 |
| rs422068 | chr14:23864804 | C/T | 0.0439 | 0.007 | 3.873e-10 |
| rs1957021 | chr14:32924505 | C/T | 0.0583 | 0.008 | 2.274e-13 |
| rs11156751 | chr14:32990437 | C/T | 0.0719 | 0.0077 | 6.941e-21 |
| rs73241997 | chr14:35173775 | T/C | 0.0733 | 0.0093 | 2.942e-15 |
| rs2738413 | chr14:64679960 | A/G | 0.0778 | 0.0067 | 2.546e-31 |
| rs74884082 | chr14:73249419 | C/T | 0.0493 | 0.0078 | 3.479e-10 |
| rs10873298 | chr14:77426525 | C/T | 0.0401 | 0.0069 | 7.072e-09 |
| rs147301839 | chr15:57924714 | C/A | 0.3328 | 0.0523 | 1.931e-10 |
| rs7170477 | chr15:64103777 | A/G | 0.0393 | 0.0072 | 4.977e-08 |
| rs745636 | chr15:70457720 | G/A | 0.0392 | 0.0122 | 7.159e-10 |
| rs74022964 | chr15:73677264 | T/C | 0.1132 | 0.009 | 3.51e-36 |
| rs12908004 | chr15:80676925 | G/A | 0.0732 | 0.009 | 4.115e-16 |
| rs2759301 | chr15:80994288 | A/G | 0.039 | 0.0067 | 5.038e-09 |
| rs4965430 | chr15:99268850 | C/G | 0.0441 | 0.0069 | 1.258e-10 |
| rs118159104 | chr16:1676804 | G/T | 0.1737 | 0.0325 | 9.275e-08 |
| rs140185678 | chr16:2003016 | A/G | 0.1659 | 0.0218 | 2.434e-14 |
| rs77316573 | chr16:2265271 | T/C | 0.0529 | 0.0089 | 3.265e-09 |
| rs2359171 | chr16:73053022 | A/T | 0.1746 | 0.0086 | 4.65e-91 |
| rs876727 | chr16:73067761 | T/G | 0.084 | 0.0084 | 1.974e-23 |
| rs7225165 | chr17:1309850 | G/A | 0.0655 | 0.0111 | 3.203e-09 |
| rs9899183 | chr17:7452977 | T/C | 0.0452 | 0.0075 | 2.017e-09 |
| rs72811294 | chr17:12618680 | G/C | 0.072 | 0.0106 | 9.67e-12 |
| rs11658278 | chr17:38031164 | T/C | 0.0443 | 0.0067 | 3.465e-11 |
| rs1563304 | chr17:44874453 | T/C | 0.0644 | 0.0092 | 2.557e-12 |
| rs12604076 | chr17:76773638 | T/C | 0.0365 | 0.0066 | 3.627e-08 |
| rs9953366 | chr18:46474192 | C/T | 0.049 | 0.0073 | 1.823e-11 |
| rs9963878 | chr18:48679522 | C/T | 0.0653 | 0.012 | 4.845e-08 |
| rs8088085 | chr18:48708548 | A/C | 0.0365 | 0.0067 | 4.794e-08 |
| rs2974231 | chr19:48170757 | A/G | 0.033 | 0.0104 | 1.107e-09 |
| rs2145274 | chr20:6572014 | A/C | 0.0169 | 0.0231 | 7.469e-12 |
| rs2834618 | chr21:36119111 | T/G | 0.0944 | 0.0112 | 3.412e-17 |
| rs56040242 | chr21:45766944 | A/G | 0.0417 | 0.0121 | 1.264e-08 |
| rs464901 | chr22:18597502 | T/C | 0.0508 | 0.0072 | 1.531e-12 |
| rs133902 | chr22:26164079 | T/C | 0.0419 | 0.0068 | 9.137e-10 |

**Table S2. Characteristics of participants at the index age of 45 years according to PRS, divided into Low, Intermediate, and High**

| **Variables** | **Low (N =21118))** | **Intermediate (N = 41910)** | **High (N = 21178)** | ***p* value** |
| --- | --- | --- | --- | --- |
| **Sex** |  |  |  | 0.0085 |
| **Female** | 11340 (53.7) | 22812 (54.4) | 11689 (55.2) |  |
| **Male** | 9778 (46.3) | 19098 (45.6) | 9489 (44.8) |  |
| **Ethnicity** |  |  |  | <.0001 |
| **Non-White** | 2026 (9.6) | 3904 (9.3) | 2390 (11.3) |  |
| **White** | 19092 (90.4) | 38006 (90.7) | 18788 (88.7) |  |
| **Smoking** |  |  |  | 0.5741 |
| **Optimal** | 12882 (61.0) | 25701 (61.3) | 12982 (61.3) |  |
| **Borderline** | 5341 (25.3) | 10400 (24.8) | 5225 (24.7) |  |
| **Elevated** | 2895 (13.7) | 5809 (13.9) | 2971 (14.0) |  |
| **Alcohol consumption** |  |  |  | 0.8844 |
| **Optimal** | 20536 (97.2) | 40730 (97.2) | 20579 (97.2) |  |
| **Elevated** | 582 (2.8) | 1180 (2.8) | 599 (2.8) |  |
| **BMI** |  |  |  | 0.2347 |
| **Optimal** | 8071 (38.2) | 16238 (38.7) | 8296 (39.2) |  |
| **Borderline** | 8487 (40.2) | 16552 (39.5) | 8301 (39.2) |  |
| **Elevated** | 4560 (21.6) | 9120 (21.8) | 4581 (21.6) |  |
| **Blood pressure** |  |  |  | 0.7420 |
| **Optimal** | 5370 (25.4) | 10534 (25.1) | 5324 (25.1) |  |
| **Borderline** | 8598 (40.7) | 17252 (41.2) | 8640 (40.8) |  |
| **Elevated** | 7150 (33.9) | 14124 (33.7) | 7214 (34.1) |  |
| **Diabetes mellitus** |  |  |  | 0.6237 |
| **Optimal** | 20475 (97.0) | 40671 (97.0) | 20567 (97.1) |  |
| **Elevated** | 643 (3.0) | 1239 (3.0) | 611 (2.9) |  |
| **Heart history** |  |  |  | 0.3339 |
| **Optimal** | 20997 (99.4) | 41677 (99.4) | 21077 (99.5) |  |
| **Elevated** | 121 (0.6) | 233 (0.6) | 101 (0.5) |  |

**Table S3. Characteristics of participants at the index age of 55 years according to PRS, divided into Low, Intermediate, and High**

| **Variables** | **Low (N = 29380)** | **Intermediate (N = 58811)** | **High (N = 29329)** | ***p* value** |
| --- | --- | --- | --- | --- |
| **Sex** |  |  |  | 0.0398 |
| **Female** | 16485 (56.1) | 33071 (56.2) | 16727 (57.0) |  |
| **Male** | 12895 (43.9) | 25740 (43.8) | 12602 (43.0) |  |
| **Ethnicity** |  |  |  | <.0001 |
| **Non-White** | 1602 (5.5) | 3133 (5.3) | 2048 (7.0) |  |
| **White** | 27778 (94.5) | 55678 (94.7) | 27281 (93.0) |  |
| **Smoking** |  |  |  | 0.4456 |
| **Optimal** | 16543 (56.3) | 33119 (56.3) | 16659 (56.8) |  |
| **Borderline** | 9633 (32.8) | 19185 (32.6) | 9421 (32.1) |  |
| **Elevated** | 3204 (10.9) | 6507 (11.1) | 3249 (11.1) |  |
| **Alcohol consumption** |  |  |  | 0.0769 |
| **Optimal** | 28510 (97.0) | 56996 (96.9) | 28504 (97.2) |  |
| **Elevated** | 870 (3.0) | 1815 (3.1) | 825 (2.8) |  |
| **BMI** |  |  |  | 0.8660 |
| **Optimal** | 9905 (33.7) | 20032 (34.1) | 9946 (33.9) |  |
| **Borderline** | 12126 (41.3) | 24171 (41.1) | 12110 (41.3) |  |
| **Elevated** | 7349 (25.0) | 14608 (24.8) | 7273 (24.8) |  |
| **Blood pressure** |  |  |  | 0.2670 |
| **Optimal** | 4258 (14.5) | 8280 (14.1) | 4094 (14.0) |  |
| **Borderline** | 9777 (33.3) | 19722 (33.5) | 9744 (33.2) |  |
| **Elevated** | 15345 (52.2) | 30809 (52.4) | 15491 (52.8) |  |
| **Diabetes mellitus** |  |  |  | 0.2322 |
| **Optimal** | 27769 (94.5) | 55736 (94.8) | 27798 (94.8) |  |
| **Elevated** | 1611 (5.5) | 3075 (5.2) | 1531 (5.2) |  |
| **Heart history** |  |  |  | 0.5814 |
| **Optimal** | 28921 (98.4) | 57839 (98.3) | 28860 (98.4) |  |
| **Elevated** | 459 (1.6) | 972 (1.7) | 469 (1.6) |  |

**Table S4. Characteristics of participants at the index age of 65 years according to PRS, divided into Low, Intermediate, and High**

| **Variables** | **Low (N = 37730)** | **Intermediate (N = 74193)** | **High (N = 35255)** | ***p* value** |
| --- | --- | --- | --- | --- |
| **Sex** |  |  |  | <.0001 |
| **Female** | 19811 (52.5) | 39220 (52.9) | 19088 (54.1) |  |
| **Male** | 17919 (47.5) | 34973 (47.1) | 16167 (45.9) |  |
| **Ethnicity** |  |  |  | <.0001 |
| **Non-White** | 1185 (3.1) | 2109 (2.8) | 1270 (3.6) |  |
| **White** | 36545 (96.9) | 72084 (97.2) | 33985 (96.4) |  |
| **Smoking** |  |  |  | 0.9599 |
| **Optimal** | 18946 (50.2) | 37404 (50.4) | 17760 (50.4) |  |
| **Borderline** | 15712 (41.6) | 30759 (41.5) | 14604 (41.4) |  |
| **Elevated** | 3072 (8.1) | 6030 (8.1) | 2891 (8.2) |  |
| **Alcohol consumption** |  |  |  | 0.0473 |
| **Optimal** | 36803 (97.5) | 72468 (97.7) | 34486 (97.8) |  |
| **Elevated** | 927 (2.5) | 1725 (2.3) | 769 (2.2) |  |
| **BMI** |  |  |  | 0.0142 |
| **Optimal** | 11357 (30.1) | 22467 (30.3) | 10977 (31.1) |  |
| **Borderline** | 17100 (45.3) | 33756 (45.5) | 15838 (44.9) |  |
| **Elevated** | 9273 (24.6) | 17970 (24.2) | 8440 (23.9) |  |
| **Blood pressure** |  |  |  | 0.2926 |
| **Optimal** | 2388 (6.3) | 4507 (6.1) | 2240 (6.4) |  |
| **Borderline** | 8657 (22.9) | 17066 (23.0) | 8026 (22.8) |  |
| **Elevated** | 26685 (70.7) | 52620 (70.9) | 24989 (70.9) |  |
| **Diabetes mellitus** |  |  |  | 0.0576 |
| **Optimal** | 34681 (91.9) | 68301 (92.1) | 32569 (92.4) |  |
| **Elevated** | 3049 (8.1) | 5892 (7.9) | 2686 (7.6) |  |
| **Heart history** |  |  |  | 0.3040 |
| **Optimal** | 36316 (96.3) | 71452 (96.3) | 34006 (96.5) |  |
| **Elevated** | 1414 (3.7) | 2741 (3.7) | 1249 (3.5) |  |

**Table S5. 10-year risk (%) of atrial fibrillation by individual risk factors, after adjustment for competing risk of death**

| **Risk factors and category** | **Index age 45 years** | |  | **Index age 55 years** | |  | **Index age 65 years** | |
| --- | --- | --- | --- | --- | --- | --- | --- | --- |
|  | **No. of atrial**  **fibrillation**  **cases/total** | **10-year risk**  **(95% CI)** |  | **No. of atrial**  **fibrillation**  **cases/total** | **10-year risk**  **(95% CI)** |  | **No. of atrial**  **fibrillation**  **cases/total** | **10-year risk**  **(95% CI)** |
| **Smoking** |  |  |  |  |  |  |  |  |
| **Optimal** | 501/51565 | 0.60 (0.53, 0.67) |  | 1848/66321 | 1.81 (1.71, 1.92) |  | 5492/74110 | 5.36 (5.19, 5.53) |
| **Borderline** | 258/20966 | 0.74 (0.62, 0.86) |  | 1484/38239 | 2.29 (2.13, 2.45) |  | 6167/61075 | 7.18 (6.97, 7.39) |
| **Elevated** | 165/11675 | 0.86 (0.68, 1.03) |  | 551/12960 | 2.51 (2.23, 2.79) |  | 1265/11993 | 8.05 (7.55, 8.56) |
| **Alcohol consumption** |  |  |  |  |  |  |  |  |
| **Optimal** | 867/81845 | 0.65 (0.59, 0.70) |  | 3678/114010 | 2.00 (1.91, 2.08) |  | 12458/143757 | 6.24 (6.11, 6.36) |
| **Elevated** | 57/2361 | 1.57 (1.04, 2.09) |  | 205/3510 | 3.63 (2.99, 4.27) |  | 466/3421 | 10.51 (9.45, 11.57) |
| **Body mass index** |  |  |  |  |  |  |  |  |
| **Optimal** | 222/32605 | 0.41 (0.34, 0.48) |  | 895/39883 | 1.37 (1.25, 1.49) |  | 2926/44801 | 4.60 (4.40, 4.80) |
| **Borderline** | 349/33340 | 0.66 (0.57, 0.75) |  | 1431/48407 | 1.87 (1.74, 2.00) |  | 5656/66694 | 6.07 (5.88, 6.26) |
| **Elevated** | 353/18261 | 1.16 (1.00, 1.32) |  | 1557/29230 | 3.25 (3.04, 3.47) |  | 4342/35683 | 9.00 (8.69, 9.31) |
| **Blood pressure** |  |  |  |  |  |  |  |  |
| **Optimal** | 118/21228 | 0.32 (0.24, 0.40) |  | 297/16632 | 1.20 (1.03, 1.37) |  | 491/9135 | 4.03 (3.62, 4.45) |
| **Borderline** | 309/34490 | 0.55 (0.47, 0.63) |  | 901/39243 | 1.49 (1.36, 1.61) |  | 1890/33749 | 4.23 (4.01, 4.45) |
| **Elevated** | 497/28488 | 1.08 (0.96, 1.21) |  | 2685/61645 | 2.63 (2.5, 2.77) |  | 10543/104294 | 7.21 (7.05, 7.37) |
| **Diabetes mellitus (type 1 or 2)** |  |  |  |  |  |  |  |  |
| **Optimal** | 844/81713 | 0.63 (0.57, 0.68) |  | 3440/111303 | 1.92 (1.84, 2.00) |  | 11363/1135551 | 6.07 (5.94, 6.2) |
| **Elevated** | 80/2493 | 2.15 (1.54, 2.75) |  | 443/6217 | 4.30 (3.78, 4.82) |  | 1561/11627 | 9.44 (8.89, 9.99) |
| **History of heart failure or myocardial infarction** | | |  |  |  |  |  |  |
| **Optimal** | 894/83751 | 0.66 (0.60, 0.71) |  | 3661/115620 | 1.96 (1.87, 2.04) |  | 11859/141774 | 6.03 (5.90, 6.15) |
| **Elevated** | 30/455 | 3.52 (1.75, 5.29) |  | 222/1900 | 7.58 (6.33, 8.83) |  | 1065/5404 | 14.43 (13.46, 15.40) |

**Table S6. 10-year risk (%) of atrial fibrillation in PRS in men according to the risk factor burden, after adjustment for competing risk of death**

| **Study sample (index age) and risk factor burdens** | **Low** | | | **Intermediate** | | | **High** | | |
| --- | --- | --- | --- | --- | --- | --- | --- | --- | --- |
|  | **No. of atrial**  **fibrillation**  **cases/total** | **10-year risk**  **(95% CI)** | **p†** | **No. of atrial**  **fibrillation**  **cases/total** | **10-year risk**  **(95% CI)** | **p†** | **No. of atrial**  **fibrillation**  **cases/total** | **10-year risk**  **(95% CI)** | **p†** |
| **45 years** |  |  |  |  |  |  |  |  |  |
| **Optimal** | 1/347 | 0.33 (0.00, 0.97) |  | 5/673 | 0.64 (0.01, 1.27) |  | 5/381 | 0.87 (0.01, 1.85) |  |
| **Borderline** | 22/3483 | 0.49 (0.25, 0.74) | 0.65 | 56/6897 | 0.50 (0.33, 0.67) | 0.68 | 78/3304 | 1.39 (0.98, 1.81) | 0.34 |
| **Elevated** | 56/5948 | 0.59 (0.39, 0.80) | 0.45 | 199/11528 | 0.92 (0.74, 1.10) | 0.41 | 202/5804 | 2.25 (1.85, 2.64) | 0.01 |
| **55 years** |  |  |  |  |  |  |  |  |  |
| **Optimal** | 2/286 | 0.35 (0.01, 1.03) |  | 19/567 | 2.06 (0.85, 3.27) |  | 9/265 | 2.86 (0.76, 4.96) |  |
| **Borderline** | 46/3225 | 0.93 (0.58, 1.28) | 0.14 | 166/6511 | 1.69 (1.37, 2.02) | 0.58 | 200/3198 | 4.66 (3.89, 5.43) | 0.12 |
| **Elevated** | 268/9354 | 1.71 (1.43, 1.98) | <0.001 | 905/18662 | 2.96 (2.70, 3.21) | 0.16 | 826/9139 | 5.93 (5.43, 6.44) | 0.005 |
| **65 years** |  |  |  |  |  |  |  |  |  |
| **Optimal** | 12/184 | 5.29 (1.92, 8.67) |  | 17/334 | 4.24 (1.97, 6.52) |  | 24/164 | 12.80 (7.55, 18.05) |  |
| **Borderline** | 123/2840 | 3.24 (2.56, 3.92) | 0.25 | 377/5687 | 5.03 (4.44, 5.62) | 0.52 | 313/2599 | 9.24 (8.09, 10.39) | 0.20 |
| **Elevated** | 1158/14895 | 5.09 (4.72, 5.45) | 0.91 | 3438/28952 | 8.56 (8.23, 8.89) | <0.001 | 2511/13404 | 14.32 (13.71, 14.94) | 0.58 |

† Test comparing lifetime risk in borderline and elevated risk groups with optimal risk group by z ratio test (that is, difference in lifetime risk between two groups divided by its standard error).

**Table S7. 10-year risk (%) of atrial fibrillation in PRS in women according to the risk factor burden, after adjustment for competing risk of death**

| **Study sample (index age) and risk factor burdens** | **Low** | | | **Intermediate** | | | **High** | | |
| --- | --- | --- | --- | --- | --- | --- | --- | --- | --- |
|  | **No. of atrial**  **fibrillation**  **cases/total** | **10-year risk**  **(95% CI)** | **p†** | **No. of atrial**  **fibrillation**  **cases/total** | **10-year risk**  **(95% CI)** | **p†** | **No. of atrial**  **fibrillation**  **cases/total** | **10-year risk**  **(95% CI)** | **p†** |
| **45 years** |  |  |  |  |  |  |  |  |  |
| **Optimal** | 4/1627 | 0.25 (0.01, 0.50) |  | 5/3231 | 0.08 (0.01, 0.19) |  | 10/1654 | 0.26 (0.01, 0.51) |  |
| **Borderline** | 8/4626 | 0.09 (0.01, 0.18) | 0.24 | 38/9159 | 0.23 (0.13, 0.33) | 0.04 | 35/4715 | 0.36 (0.19, 0.54) | 0.50 |
| **Elevated** | 34/5087 | 0.47 (0.28, 0.66) | 0.18 | 84/10422 | 0.56 (0.40, 0.71) | <0.001 | 82/5320 | 0.99 (0.71, 1.27) | <0.001 |
| **55 years** |  |  |  |  |  |  |  |  |  |
| **Optimal** | 7/1158 | 0.45 (0.06, 0.85) |  | 22/2261 | 0.70 (0.35, 1.06) |  | 29/1183 | 1.94 (1.13, 2.74) |  |
| **Borderline** | 34/5610 | 0.42 (0.25, 0.60) | 0.91 | 128/11267 | 0.64 (0.48, 0.79) | 0.74 | 124/5564 | 1.28 (0.98, 1.59) | 0.14 |
| **Elevated** | 147/9717 | 0.87 (0.68, 1.06) | 0.06 | 516/19543 | 1.44 (1.26, 1.61) | <0.001 | 435/9981 | 2.64 (2.31, 2.97) | 0.11 |
| **65 years** |  |  |  |  |  |  |  |  |  |
| **Optimal** | 12/592 | 1.33 (0.34, 2.32) |  | 18/1046 | 0.91 (0.32, 1.50) |  | 28/547 | 4.00 (2.32, 5.68) |  |
| **Borderline** | 130/4701 | 1.94 (1.54, 2.35) | 0.26 | 316/9369 | 2.48 (2.15, 2.80) | <0.001 | 292/4490 | 4.68 (4.04, 5.32) | 0.47 |
| **Elevated** | 593/14518 | 2.71 (2.43, 2.98) | 0.008 | 2010/28805 | 4.86 (4.60, 5.12) | <0.001 | 1552/14051 | 8.02 (7.55, 8.48) | <0.001 |

† Test comparing lifetime risk in borderline and elevated risk groups with optimal risk group by z ratio test (that is, difference in lifetime risk between two groups divided by its standard error).

**Table S8. Attributable proportion of risk factor burdens and PRS and AF incidence in overall and by sex**

| **Study sample (index age) and risk category** | **Attributable proportion (AR)†‡** | | | | | |
| --- | --- | --- | --- | --- | --- | --- |
|  | **All** | | **Men** | | **Women** | |
|  | **Intermediate** | **High** | **Intermediate** | **High** | **Intermediate** | **High** |
| **45 years** |  |  |  |  |  |  |
| **Optimal** |  |  |  |  |  |  |
| **Borderline** | 0.36 (-0.29, 1.02) | 0.28 (-0.17, 0.73) | -0.34 (-1.51, 0.83) | 0.29 (-0.30, 0.88) | 0.81 (0.07, 1.55) | 0.28 (-0.39, 0.95) |
| **Elevated** | 0.37 (0.05, 0.69) | 0.44 (0.20, 0.68) | 0.18 (-0.36, 0.73) | 0.43 (0.04, 0.82) | 0.29 (-0.18, 0.76) | 0.33 (-0.03, 0.69) |
| **55 years** |  |  |  |  |  |  |
| **Optimal** |  |  |  |  |  |  |
| **Borderline** | -0.16 (-0.64, 0.32) | 0.08 (-0.24, 0.41) | -0.63 (-1.40, 0.15) | 0.34 (-0.06, 0.75) | 0.12 (-0.48, 0.72) | -0.12 (-0.63, 0.38) |
| **Elevated** | 0.13 (-0.08, 0.35) | 0.29 (0.10, 0.48) | -0.16 (-0.55, 0.24) | 0.39 (0.11, 0.67) | 0.28 (0.01, 0.54) | 0.22 (-0.04 ,0.47) |
| **65 years** |  |  |  |  |  |  |
| **Optimal** |  |  |  |  |  |  |
| **Borderline** | 0.40 (0.02, 0.78) | 0.07 (-0.24, 0.38) | 0.55 (-0.14, 1.24) | -0.06 (-0.66, 0.54) | 0.27 (-0.18, 0.72) | 0.11 (-0.25, 0.48) |
| **Elevated** | 0.45 (0.26, 0.65) | 0.28 (0.09, 0.47) | 0.48 (0.09, 0.86) | 0.15 (-0.22, 0.53) | 0.46 (0.25, 0.68) | 0.36 (0.15, 0.57) |

†All results were calculated after adjusting for sex.

‡: The estimates of AR were calculated based on the reference group with low level of risk factor profile (Optimal 0/0) and low level of PRS (Low).

**Table S9. Combined effects of risk factor burdens and PRS and AF incidence in men**

| **Study sample (index age) and risk factor burdens** | **PRS levels (HR, 95% CI)** | | |  | **RERI** | | **p for**  **interaction §** |
| --- | --- | --- | --- | --- | --- | --- | --- |
|  | **Low** | **Intermediate** | **High** |  | **Intermediate** | **High** |  |
| **45 years** |  |  |  |  |  |  |  |
| **Optimal** | 1.00 | 2.59 (0.30, 22.17) | 4.63 (0.54, 39.62) |  |  |  | 0.44 |
| **Borderline** | 2.16 (0.29, 16.03) | 2.80 (0.39, 20.19) | 8.16 (1.14, 58.62)* |  | -0.95 (-5.55, 3.64) | 2.36 (-2.90, 7.61) |  |
| **Elevated** | 3.19 (0.44, 23.01) | 5.85 (0.82, 41.74) | 11.93 (1.67, 85.09)* |  | 1.07 (-1.48, 3.63) | 5.12 (-4.09, 14.34) |  |
| **55 years** |  |  |  |  |  |  |  |
| **Optimal** | 1.00 | 4.88 (1.14, 20.92)* | 4.94 (1.07, 22.83)* |  |  |  | 0.02 |
| **Borderline** | 2.00 (0.49, 8.24) | 3.62 (0.90, 14.59) | 9.07 (2.26, 36.43)* |  | -2.26 (-7.33, 2.81) | 3.12 (-1.45, 7.70) |  |
| **Elevated** | 3.97 (0.99, 15.93) | 6.80 (1.70, 27.18)* | 12.99 (3.25, 51.93)* |  | -1.06 (-4.71, 2.59) | 5.06 (-1.48, 11.60) |  |
| **65 years** |  |  |  |  |  |  |  |
| **Optimal** | 1.00 | 0.80 (0.38, 1.67) | 2.36 (1.18, 4.72)* |  |  |  | 0.12 |
| **Borderline** | 0.65 (0.36, 1.17) | 1.00 (0.57, 1.78) | 1.89 (1.07, 3.36)* |  | 0.55 (0.07, 1.03) | -0.12 (-1.28, 1.04) |  |
| **Elevated** | 1.14 (0.65, 2.00) | 1.79 (1.02, 3.14)* | 2.95 (1.68, 5.18)* |  | 0.84 (0.45, 1.25)* | 0.45 (-0.56, 1.46) |  |

‡: The estimates of RERI were calculated based on the reference group with optimal risk factor burdens and low PRS.

§: Likelihood tests were applied to test the significance of the interaction term by comparing the model with and without the interaction term.

*: P-values < 0.05, and the P-values for additive scale in each index age were adjusted by FDR (False Discovery Rate).

Abbreviations: HR, hazard ratio; CI, confidence interval; RERI, relative excess risk due to interaction

**Table S10**. **Combined effects of risk factor burdens and PRS and AF incidence in women**

| **Study sample (index age) and risk factor burdens** | **PRS levels (HR, 95% CI)** | | |  | **RERI** | | **p for**  **interaction §** |
| --- | --- | --- | --- | --- | --- | --- | --- |
|  | **Low** | **Intermediate** | **High** |  | **Intermediate** | **High** |  |
| **45 years** |  |  |  |  |  |  |  |
| **Optimal** | 1.00 | 0.63 (0.17, 2.34) | 2.47 (0.77, 7.86) |  |  |  | 0.28 |
| **Borderline** | 0.69 (0.21, 2.30) | 1.66 (0.59, 4.66) | 3.00 (1.07, 8.43)* |  | 1.34 (0.38, 2.31)* | 0.83 (-1.06, 2.72) |  |
| **Elevated** | 2.64 (0.94, 7.42) | 3.19 (1.17, 8.69)* | 6.11 (2.24, 16.66)* |  | 0.93 (-0.32, 2.18) | 2.01 (-0.41, 4.44) |  |
| **55 years** |  |  |  |  |  |  |  |
| **Optimal** | 1.00 | 1.64 (0.70, 3.83) | 4.10 (1.80, 9.34)* |  |  |  | 0.46 |
| **Borderline** | 0.98 (0.43, 2.20) | 1.83 (0.86, 3.92) | 3.62 (1.69, 7.74)* |  | 0.22 (-0.79, 1.22) | -0.44 (-2.41, 1.53) |  |
| **Elevated** | 2.38 (1.11, 5.07)* | 4.18 (1.99, 8.81)* | 6.98 (3.31, 14.72)* |  | 1.16 (0.29, 2.03)* | 1.53 (-0.19, 3.25) |  |
| **65 years** |  |  |  |  |  |  |  |
| **Optimal** | 1.00 | 0.86 (0.42, 1.78) | 2.50 (1.27, 4.91)* |  |  |  | 0.005 |
| **Borderline** | 1.32 (0.74, 2.39) | 1.62 (0.91, 2.88) | 3.18 (1.79, 5.65)* |  | 0.44 (-0.15, 1.02) | 0.36 (-0.73, 1.44) |  |
| **Elevated** | 1.89 (1.07, 3.33)* | 3.26 (1.85, 5.73)* | 5.30 (3.01, 9.33)* |  | 1.51 (0.98, 2.04)* | 1.92 (0.81, 3.02)* |  |

‡: The estimates of RERI were calculated based on the reference group with optimal risk factor burdens and low PRS.

§: Likelihood tests were applied to test the significance of the interaction term by comparing the model with and without the interaction term.

*: P-values < 0.05, and the P-values for additive scale in each index age were adjusted by FDR (False Discovery Rate).

Abbreviations: HR, hazard ratio; CI, confidence interval; RERI, relative excess risk due to interaction

**Table S11. Distribution of risk factor profiles**

| **Risk factor**  **profiles** | **No.**  **of elevated**  **risk factors** | **No.**  **of borderline**  **risk factors** | **45 years (N = 84206)** | **55 years (N = 117520)** | **65 years (N = 147178)** |
| --- | --- | --- | --- | --- | --- |
| **Optimal** | 0 | 0 | 7913 (9.40) | 5719 (4.87) | 2867 (1.95) |
| **Borderline** | 0 | 1 | 15231 (18.09) | 14992 (12.76) | 11038 (7.50) |
|  | 0 | 2 | 13356 (15.86) | 15226 (12.96) | 12947 (8.80) |
|  | 0 | >= 3 | 3597 (4.27) | 5187 (4.41) | 5701 (3.87) |
| **Elevated** | 1 | any | 28166 (33.45) | 45475 (38.70) | 69283 (47.07) |
|  | 2 | any | 12689 (15.07) | 23820 (20.27) | 34484 (23.43) |
|  | 3 | any | 3254 (3.86) | 7101 (6.04) | 10858 (7.38) |

**Table S12. Additive and multiplicative interactions between risk factor profiles and PRS in relation to AF incidence**

| **Study sample (index age) and PRS** | **RERI†‡** | | | | | | | **p for**  **interaction §** |
| --- | --- | --- | --- | --- | --- | --- | --- | --- |
|  | **Borderline** | | |  | **Elevated** | | |  |
|  | **0/1** | **0/2** | **0/>= 3** |  | **1/any** | **2/any** | **>= 3/any** |  |
| **45 years** |  |  |  |  |  |  |  |  |
| **Intermediate** | 0.34 (-0.83, 1.51) | 0.85 (-0.24, 1.94) | 0.99 (-0.71, 2.69) |  | 0.77 (-0.19, 1.73) | 2.83 (0.53, 5.12)* | 1.90 (-2.04, 5.84) | 0.82 |
| **High** | 0.75 (-1.17, 2.68) | 1.89 (-0.37, 4.16) | 1.12 (-1.51, 3.76) |  | 1.18 (-0.63, 3.00) | 6.27 (0.88, 11.66)* | 12.78 (-0.05, 25.61) |  |
| **55 years** |  |  |  |  |  |  |  |  |
| **Intermediate** | -0.25 (-1.49, 0.98) | -0.50 (-1.85, 0.84) | -0.19 (-1.60, 1.23) |  | 0.13 (-0.87, 1.14) | 1.22 (0.16, 2.28)* | 2.47 (0.28, 4.66)* | 0.03 |
| **High** | -0.27 (-2.11, 1.57) | 0.58 (-1.11, 2.26) | 2.24 (-0.10, 4.58) |  | 1.48 (-0.07, 3.03) | 3.55 (1.12, 5.97)* | 6.97 (2.11, 11.83)* |  |
| **65 years** |  |  |  |  |  |  |  |  |
| **Intermediate** | 0.52 (0.10, 0.94)* | 0.53 (0.13, 0.93)* | 0.54 (0.06, 1.02)* |  | 1.03 (0.73, 1.32)* | 1.35 (0.98, 1.72)* | 1.32 (0.79, 1.85)* | <0.001 |
| **High** | 0.10 (-0.75, 0.94) | 0.09 (-0.73, 0.92) | 0.40 (-0.49, 1.29) |  | 0.85 (0.16, 1.53)* | 1.72 (0.94, 2.51)* | 1.93 (0.91, 2.94)* |  |

†All results were calculated after adjusting for sex.

‡: The estimates of RERI were calculated based on the reference group with low level of risk factor profile (Optimal 0/0) and low level of PRS (Low).

§: Likelihood tests were applied to test the significance of the interaction term by comparing the model with and without the interaction term.

*: P-values < 0.05

Abbreviations: RERI, relative excess risk due to interaction

**Table S13. 10-year risk (%) of atrial fibrillation in men by risk factor profiles (number of elevated/borderline risk factors) and PRS, after adjustment for competing risk of death**

| **Risk factor profile and**  **number of elevated/**  **borderline risk factors** | **PRS** | | | | | | | |
| --- | --- | --- | --- | --- | --- | --- | --- | --- |
|  | **Low** | |  | **Intermediate** | |  | **High** | |
|  | **No. of atrial**  **fibrillation**  **cases/total** | **10-year risk**  **(95% CI)** |  | **No. of atrial**  **fibrillation**  **cases/total** | **10-year risk**  **(95% CI)** |  | **No. of atrial**  **fibrillation**  **cases/total** | **10-year risk**  **(95% CI)** |
| **Index age 45 years** |  |  |  |  |  |  |  |  |
| **Optimal** |  |  |  |  |  |  |  |  |
| **0/0** | 1/347 | 0.33 (0.00, 0.97) |  | 5/673 | 0.64 (0.01, 1.27) |  | 5/381 | 0.87 (0.00, 1.85) |
| **Borderline** |  |  |  |  |  |  |  |  |
| **0/1** | 10/1251 | 0.72 (0.22, 1.22) |  | 15/2493 | 0.29 (0.08, 0.51) |  | 27/1153 | 1.51 (0.77, 2.25) |
| **0/2** | 10/1680 | 0.43 (0.11, 0.75) |  | 33/3332 | 0.62 (0.34, 0.90) |  | 39/1646 | 1.17 (0.63, 1.72) |
| **0/>=3** | 2/552 | 0.18 (0.00, 0.54) |  | 8/1072 | 0.59 (0.12, 1.05) |  | 12/505 | 1.83 (0.64, 3.01) |
| **Elevated** |  |  |  |  |  |  |  |  |
| **1/any** | 26/3632 | 0.39 (0.18, 0.61) |  | 86/6924 | 0.66 (0.46, 0.85) |  | 82/3508 | 1.66 (1.21, 2.10) |
| **2/any** | 18/1792 | 0.73 (0.32, 1.15) |  | 76/3549 | 1.24 (0.86, 1.63) |  | 80/1747 | 2.62 (1.84, 3.40) |
| **>=3/any** | 12/524 | 1.49 (0.38, 2.61) |  | 37/1055 | 1.53 (0.75, 2.30) |  | 40/549 | 4.83 (2.92, 6.73) |
| **Index age 55 years** |  |  |  |  |  |  |  |  |
| **Optimal** |  |  |  |  |  |  |  |  |
| **0/0** | 2/286 | 0.35 (0.00, 1.03) |  | 19/567 | 2.06 (0.85, 3.27) |  | 9/265 | 2.86 (0.76, 4.96) |
| **Borderline** |  |  |  |  |  |  |  |  |
| **0/1** | 13/1075 | 0.69 (0.18, 1.20) |  | 59/2177 | 1.46 (0.94, 1.97) |  | 53/1071 | 4.19 (2.91, 5.46) |
| **0/2** | 26/1537 | 1.25 (0.66, 1.85) |  | 78/3098 | 1.81 (1.32, 2.30) |  | 100/1510 | 4.73 (3.61, 5.85) |
| **0/>=3** | 7/643 | 0.56 (0.00, 1.20) |  | 29/1236 | 1.80 (1.03, 2.56) |  | 47/617 | 5.28 (3.43, 7.13) |
| **Elevated** |  |  |  |  |  |  |  |  |
| **1/any** | 93/5102 | 0.97 (0.69, 1.25) |  | 345/10429 | 2.02 (1.74, 2.31) |  | 343/5057 | 4.63 (4.02, 5.24) |
| **2/any** | 98/3152 | 1.85 (1.36, 2.34) |  | 342/5957 | 3.48 (2.99, 3.96) |  | 298/2996 | 6.66 (5.73, 7.59) |
| **>= 3/any** | 77/1100 | 4.67 (3.35, 5.99) |  | 218/2276 | 5.87 (4.86, 6.88) |  | 185/1086 | 9.90 (8.07, 11.74) |
| **Index age 65 years** |  |  |  |  |  |  |  |  |
| **Optimal** |  |  |  |  |  |  |  |  |
| **0/0** | 12/184 | 5.29 (1.92, 8.67) |  | 17/334 | 4.24 (1.97, 6.52) |  | 24/164 | 12.80 (7.55, 18.05) |
| **Borderline** |  |  |  |  |  |  |  |  |
| **0/1** | 33/835 | 3.14 (1.93, 4.36) |  | 109/1694 | 4.80 (3.74, 5.87) |  | 86/783 | 9.14 (7.04, 11.23) |
| **0/2** | 56/1294 | 3.60 (2.54, 4.66) |  | 177/2535 | 5.17 (4.28, 6.07) |  | 137/1152 | 8.52 (6.85, 10.19) |
| **0/>= 3** | 34/711 | 2.72 (1.47, 3.96) |  | 91/1458 | 5.04 (3.88, 6.20) |  | 90/664 | 10.60 (8.19, 13.01) |
| **Elevated** |  |  |  |  |  |  |  |  |
| **1/any** | 479/8368 | 3.60 (3.19, 4.01) |  | 1564/16104 | 6.82 (6.42, 7.22) |  | 1230/7570 | 12.18 (11.42, 12.95) |
| **2/any** | 414/4593 | 6.06 (5.34, 6.77) |  | 1214/9199 | 9.66 (9.04, 10.29) |  | 877/4232 | 15.87 (14.73, 17.01) |
| **>= 3/any** | 265/1934 | 9.19 (7.87, 10.51) |  | 660/3649 | 13.40 (12.26, 14.55) |  | 404/1602 | 20.26 (18.23, 22.30) |

**Table S14. 10-year risk (%) of atrial fibrillation in women by risk factor profiles (number of elevated/borderline risk factors) and PRS, after adjustment for competing risk of death**

| **Risk factor profile and**  **number of elevated/**  **borderline risk factors** | **PRS** | | | | | | | |
| --- | --- | --- | --- | --- | --- | --- | --- | --- |
|  | **Low** | |  | **Intermediate** | |  | **High** | |
|  | **No. of atrial**  **fibrillation**  **cases/total** | **10-year risk**  **(95% CI)** |  | **No. of atrial**  **fibrillation**  **cases/total** | **10-year risk**  **(95% CI)** |  | **No. of atrial**  **fibrillation**  **cases/total** | **10-year risk**  **(95% CI)** |
| **Index age 45 years** |  |  |  |  |  |  |  |  |
| **Optimal** |  |  |  |  |  |  |  |  |
| **0/0** | 4/1627 | 0.25 (0.01, 0.50) |  | 5/3231 | 0.08 (0.00, 0.19) |  | 10/1654 | 0.26 (0.01, 0.51) |
| **Borderline** |  |  |  |  |  |  |  |  |
| **0/1** | 3/2590 | 0.04 (0.00, 0.13) |  | 19/5124 | 0.19 (0.07, 0.32) |  | 16/2620 | 0.40 (0.15, 0.65) |
| **0/2** | 4/1644 | 0.19 (0.00, 0.41) |  | 15/3335 | 0.22 (0.06, 0.39) |  | 18/1719 | 0.33 (0.04, 0.63) |
| **0/>= 3** | 1/392 | - |  | 4/700 | 0.58 (0.01, 1.15) |  | 1/376 | 0.28 (0.00, 0.81) |
| **Elevated** |  |  |  |  |  |  |  |  |
| **1/any** | 17/3395 | 0.39 (0.18, 0.61) |  | 36/7107 | 0.37 (0.22, 0.51) |  | 34/3600 | 0.47 (0.23, 0.71) |
| **2/any** | 11/1429 | 0.36 (0.04, 0.68) |  | 37/2728 | 0.92 (0.54, 1.31) |  | 33/1444 | 1.92 (1.18, 2.66) |
| **>= 3/any** | 6/263 | 1.95 (0.26, 3.65) |  | 11/587 | 1.14 (0.21, 2.06) |  | 15/276 | 2.91 (0.92, 4.89) |
| **Index age 55 years** |  |  |  |  |  |  |  |  |
| **Optimal** |  |  |  |  |  |  |  |  |
| **0/0** | 7/1158 | 0.45 (0.06, 0.85) |  | 22/2261 | 0.70 (0.35, 1.06) |  | 29/1182 | 1.94 (1.13, 2.74) |
| **Borderline** |  |  |  |  |  |  |  |  |
| **0/1** | 16/2632 | 0.34 (0.09, 0.59) |  | 58/5362 | 0.64 (0.41, 0.86) |  | 53/2675 | 1.12 (0.71, 1.54) |
| **0/2** | 13/2332 | 0.34 (0.09, 0.59) |  | 51/4554 | 0.57 (0.34, 0.80) |  | 50/2195 | 1.26 (0.78, 1.75) |
| **0/>= 3** | 5/646 | 0.32 (0.00, 0.75) |  | 19/1351 | 0.83 (0.34, 1.32) |  | 21/694 | 1.96 (0.90, 3.01) |
| **Elevated** |  |  |  |  |  |  |  |  |
| **1/any** | 66/6190 | 0.61 (0.40, 0.82) |  | 233/12354 | 1.07 (0.88, 1.26) |  | 222/6343 | 2.09 (1.72, 2.46) |
| **2/any** | 65/2887 | 1.33 (0.89, 1.76) |  | 199/5856 | 1.76 (1.40, 2.11) |  | 158/2972 | 3.30 (2.62, 3.97) |
| **>= 3/any** | 16/640 | 1.33 (0.41, 2.25) |  | 844/1333 | 3.47 (2.46, 4.48) |  | 55/666 | 4.99 (3.26, 6.71) |
| **Index age 65 years** |  |  |  |  |  |  |  |  |
| **Optimal** |  |  |  |  |  |  |  |  |
| **0/0** | 12/592 | 1.33 (0.34, 2.32) |  | 18/1046 | 0.91 (0.32, 1.50) |  | 28/547 | 4.00 (2.32, 5.68) |
| **Borderline** |  |  |  |  |  |  |  |  |
| **0/1** | 54/1931 | 2.03 (1.38, 2.69) |  | 134/3928 | 2.42 (1.92, 2.92) |  | 121/1867 | 4.67 (3.68, 5.67) |
| **0/2** | 54/2023 | 1.84 (1.24, 2.45) |  | 122/3999 | 2.32 (1.84, 2.80) |  | 121/1944 | 4.49 (3.53, 5.45) |
| **0/>= 3** | 22/747 | 1.98 (0.95, 3.01) |  | 60/1442 | 3.07 (2.15, 3.98) |  | 50/679 | 5.27 (3.54, 7.00) |
| **Elevated** |  |  |  |  |  |  |  |  |
| **1/any** | 308/9357 | 2.30 (1.99, 2.62) |  | 1061/18762 | 3.82 (3.53, 4.10) |  | 818/9122 | 6.49 (5.96, 7.01) |
| **2/any** | 200/4222 | 2.89 (2.36, 3.41) |  | 719/8166 | 6.29 (5.74, 6.84) |  | 573/4072 | 10.18 (9.22, 11.14) |
| **>= 3/any** | 85/939 | 5.91 (4.35, 7.47) |  | 230/1877 | 9.08 (7.74, 10.42) |  | 161/857 | 14.05 (11.65, 16.45) |

**Table S15. Multivariable prediction model of the 10-year risk of atrial fibrillation at index age 45 years**

|  | Coef. | SE(Coef.) | Covariance | p-value |
| --- | --- | --- | --- | --- |
| Male vs. Female | 0.7709 | 0.0734 |  | 0.0000 |
| White vs. Non-white | 0.6342 | 0.1415 |  | 0.0000 |
| Past smoker vs. never smoker | 0.1346 | 0.0769 |  | 0.0800 |
| Current smoker vs. never smoker | 0.2452 | 0.0909 |  | 0.0070 |
| Body mass index (kg/m2) | 0.0652 | 0.0061 |  | 0.0000 |
| Hypertension* | 0.8476 | 0.1817 | -0.0036 | 0.0000 |
| Hypertension x t | -0.0476 | 0.0225 |  | 0.0350 |
| Elevated alcohol vs. optimal alcohol | 0.4846 | 0.1395 |  | 0.0005 |
| History of MI or HF | 0.9097 | 0.1923 |  | 0.0000 |
| Diastolic blood pressure (mm Hg) | -0.0258 | 0.0058 |  | 0.0000 |
| Systolic blood pressure (mm Hg) | 0.0162 | 0.0035 |  | 0.0000 |
| Elevated diabetes vs. optimal diabetes | 0.4720 | 0.1266 |  | 0.0002 |
| Borderline PRS vs. optimal PRS | 0.4570 | 0.1027 |  | 0.0000 |
| Elevated PRS vs. optimal PRS | 1.2248 | 0.1022 |  | 0.0000 |

We entered the 11 covariates into the model without performing any variable selection.

PRS: polygenic risk score. Coef.: coefficient; SE(Coef): standard error of the coefficient. The sub-distribution hazard ratio is exp(Coef) and the associated 95% CI is from exp[Coef-1.96*SE(Coef)] to exp[Coef+1.96*SE(Coef)].

*Treatment or history of hypertension

**Table S16. Multivariable prediction model of the 10-year risk of atrial fibrillation at index age 55 years**

|  | Coef. | SE(Coef.) | Covariance | p-value |
| --- | --- | --- | --- | --- |
| Male vs. Female | 0.9103 | 0.0799 | -0.0007 | 0.0000 |
| Male vs. Female x t | -0.0322 | 0.0102 |  | 0.0016 |
| White vs. Non-white | 0.5723 | 0.0886 |  | 0.0000 |
| Past smoker vs. never smoker | 0.1474 | 0.0352 |  | 0.0000 |
| Current smoker vs. never smoker | 0.2802 | 0.0491 |  | 0.0000 |
| Body mass index (kg/m2) | 0.0595 | 0.0032 |  | 0.0000 |
| Hypertension* | 0.5362 | 0.0368 |  | 0.0000 |
| Elevated alcohol vs. optimal alcohol | 0.3332 | 0.0724 |  | 0.0000 |
| History of MI or HF | 0.5457 | 0.0738 |  | 0.0000 |
| Diastolic blood pressure (mm Hg) | -0.0179 | 0.0026 |  | 0.0000 |
| Systolic blood pressure (mm Hg) | 0.0039 | 0.0024 | -0.00000054 | 0.1100 |
| Systolic blood pressure (mm Hg) x t | 0.0006 | 0.0003 |  | 0.0380 |
| Elevated diabetes vs. optimal diabetes | 0.1714 | 0.0549 |  | 0.0018 |
| Borderline PRS vs. optimal PRS | 0.5608 | 0.0504 |  | 0.0000 |
| Elevated PRS vs. optimal PRS | 1.2187 | 0.0509 |  | 0.0000 |
|  |  |  |  |  |

PRS: polygenic risk score. Coef.: coefficient; SE(Coef): standard error of the coefficient. t: natural logarithm of time (years since index age 55 years). The subdistribution hazard ratio is exp(Coef) and the associated 95% confidence interval is from exp[Coef-1.96*SE(Coef)] to exp[Coef+1.96*SE(Coef)].

*Treatment or history of hypertension

**Table S17. Multivariable prediction model of the 10-year risk of atrial fibrillation at index age 65 years**

|  | Coef. | SE(Coef.) | Covariance | p-value |
| --- | --- | --- | --- | --- |
| Male vs. Female | 0.7258 | 0.0420 | -0.00021 | 0.0000 |
| Male vs. Female x t | -0.0297 | 0.0057 |  | 0.0000 |
| White vs. Non-white | 0.3252 | 0.0581 |  | 0.0000 |
| Past smoker vs. never smoker | 0.1211 | 0.0189 |  | 0.0000 |
| Current smoker vs. never smoker | 0.2438 | 0.0315 |  | 0.0000 |
| Body mass index (kg/m2) | 0.0459 | 0.0020 |  | 0.0000 |
| Hypertension | 0.5655 | 0.0418 | -0.00021 | 0.0000 |
| Hypertension x t | -0.0163 | 0.0056 |  | 0.0039 |
| Elevated alcohol vs. optimal alcohol | 0.2586 | 0.0475 |  | 0.0000 |
| History of MI or HF | 0.3893 | 0.0339 |  | 0.0000 |
| Diastolic blood pressure (mm Hg) | -0.0045 | 0.0028 | -0.00000097 | 0.1200 |
| Diastolic blood pressure (mm Hg) x t | -0.0016 | 0.0004 |  | 0.0000 |
| Systolic blood pressure (mm Hg) | -0.0025 | 0.0015 | -0.00000026 | 0.0940 |
| Systolic blood pressure (mm Hg) x t | 0.0012 | 0.0002 |  | 0.0000 |
| Elevated diabetes vs. optimal diabetes | 0.0178 | 0.0285 |  | 0.5300 |
| Borderline PRS vs. optimal PRS | 0.5586 | 0.0592 | -0.00042 | 0.0000 |
| Borderline PRS vs. optimal PRS x t | -0.0143 | 0.0079 |  | 0.0710 |
| Elevated PRS vs. optimal PRS | 1.2194 | 0.0608 | -0.00045 | 0.0000 |
| Elevated PRS vs. optimal PRS x t | -0.0352 | 0.0082 |  | 0.0000 |

*Treatment or history of hypertension
