# Supplementary material for: Associations of risk factor burden and genetic predisposition with the 10-year risk of atrial fibrillation: observations from a large prospective study of 348,904 participants
Source: BMC Med. 2023 Mar 8;21:88. doi: 10.1186/s12916-023-02798-7 (PMC9993634; doi:10.1186/s12916-023-02798-7)
Supplement: Supplementary file 2 — Additional file 2: Central illustration. Figure S1. Cumulative risk (%) for atrial fibrillation according to risk factor burdens (optimal, borderline, or elevated) at the index age of 45 years. Figure S2. Cumulative risk (%) for atrial fibrillation according to risk factor burdens (optimal, borderline, or elevated) at the index age of 65 years. Figure S3. Predicted 10-year risk (%) of atrial fibrillation at index age 45 years across 16 risk profiles, in men with different polygenic risk score (low, intermediate, or high). Figure S4. Predicted 10-year risk (%) of atrial fibrillation at index age 45 years across 16 risk profiles, in women with different polygenic risk score (low, intermediate, or high). Figure S5. Predicted 10-year risk (%) of atrial fibrillation at index age of 65 years across 16 risk profiles, in men with different polygenic risk score (low, intermediate, or high). Figure S6. Predicted 10-year risk (%) of atrial fibrillation at index age of 65 years across 16 risk profiles, in women with different polygenic risk score (low, intermediate, or high). Figure S7. Calibration plot of the prediction model for predicting 10-year risk at index age of 45 years. Figure S8. Calibration plot of the prediction model for predicting 10-year risk at index age 55 years. Figure S9. Calibration plot of the prediction model for predicting 10-year risk at index age 65 years. [file 12916_2023_2798_MOESM2_ESM.docx]

**Additional File 2**

**Associations of risk factor burden and genetic predisposition with the 10-year risk of atrial fibrillation: Observations from a large prospective study of 348,904 participants**

Junguo Zhang PhD, Ge Chen PhD, Xiaojie Wang PhD, Zhengmin (Min) Qian PhD, Miao Cai PhD, Michael G. Vaughn PhD, Elizabeth Bingheim PhD, Haitao Li PhD, Yanhui Gao PhD, Gregory Y. H. Lip^+^ MD, Hualiang Lin^+*^ PhD

[^+^Joint senior authors]

*Corresponding author

| Contents | | |
| --- | --- | --- |
|  | Central illustration | Page 3 |
| Figure S1 | Cumulative risk (%) for atrial fibrillation according to risk factor burdens (optimal, borderline, or elevated) at the index age of 45 years. | Page 4 |
| Figure S2 | Cumulative risk (%) for atrial fibrillation according to risk factor burdens (optimal, borderline, or elevated) at the index age of 65 years. | Page 5 |
| Figure S3 | Predicted 10-year risk (%) of atrial fibrillation at index age 45 years across 16 risk profiles, in men with different polygenic risk score (low, intermediate, or high). | Page 6 |
| Figure S4 | Predicted 10-year risk (%) of atrial fibrillation at index age 45 years across 16 risk profiles, in women with different polygenic risk score (low, intermediate, or high). | Page 7 |
| Figure S5 | Predicted 10-year risk (%) of atrial fibrillation at index age of 65 years across 16 risk profiles, in men with different polygenic risk score (low, intermediate, or high). | Page 8 |
| Figure S6 | Predicted 10-year risk (%) of atrial fibrillation at index age of 65 years across 16 risk profiles, in women with different polygenic risk score (low, intermediate, or high). | Page 9 |
| Figure S7 | Calibration plot of the prediction model for predicting 10-year risk at index age of 45 years | Page 10 |
| Figure S8 | Calibration plot of the prediction model for predicting 10-year risk at index age 55 years | Page 11 |
| Figure S9 | Calibration plot of the prediction model for predicting 10-year risk at index age 65 years | Page 12 |


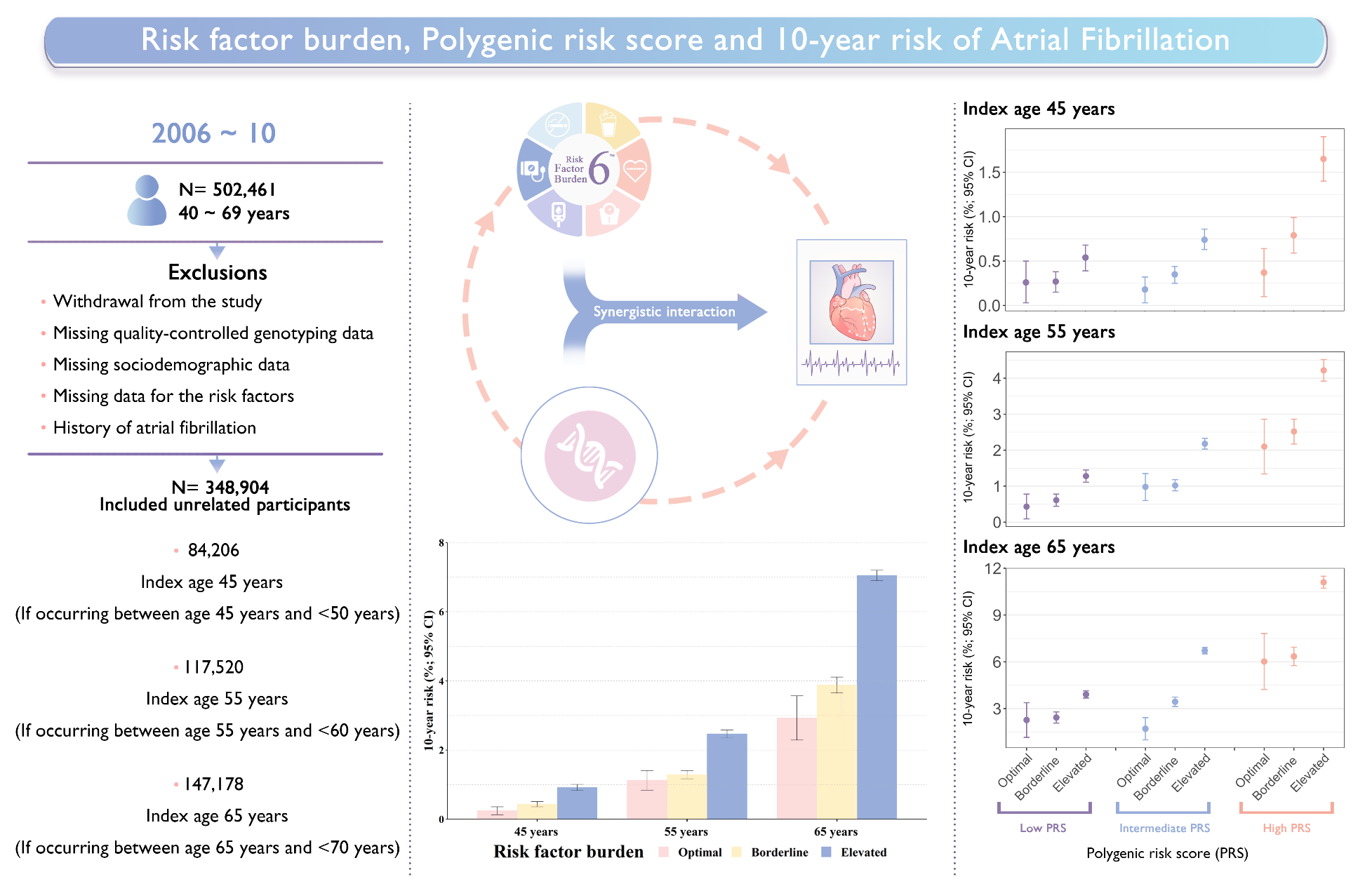


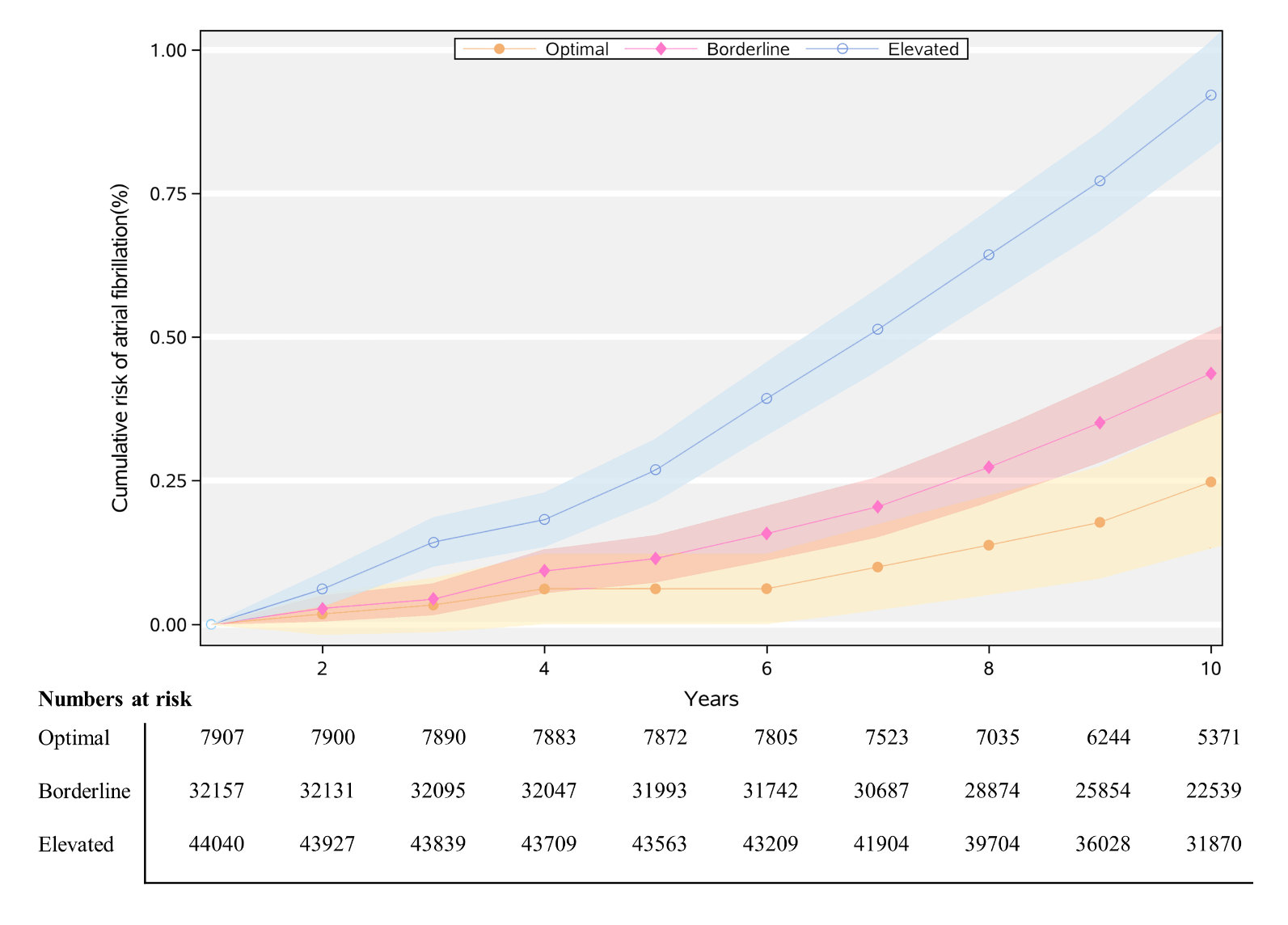


**Figure S1.** Cumulative risk (%) for atrial fibrillation according to risk factor burdens (optimal, borderline, or elevated) at the index age of 45 years. Shading = 95% CI. Participants entered the study sample between ages 45 and < 50 years; therefore, the number at risk increased from age 45 years to < 50 years.


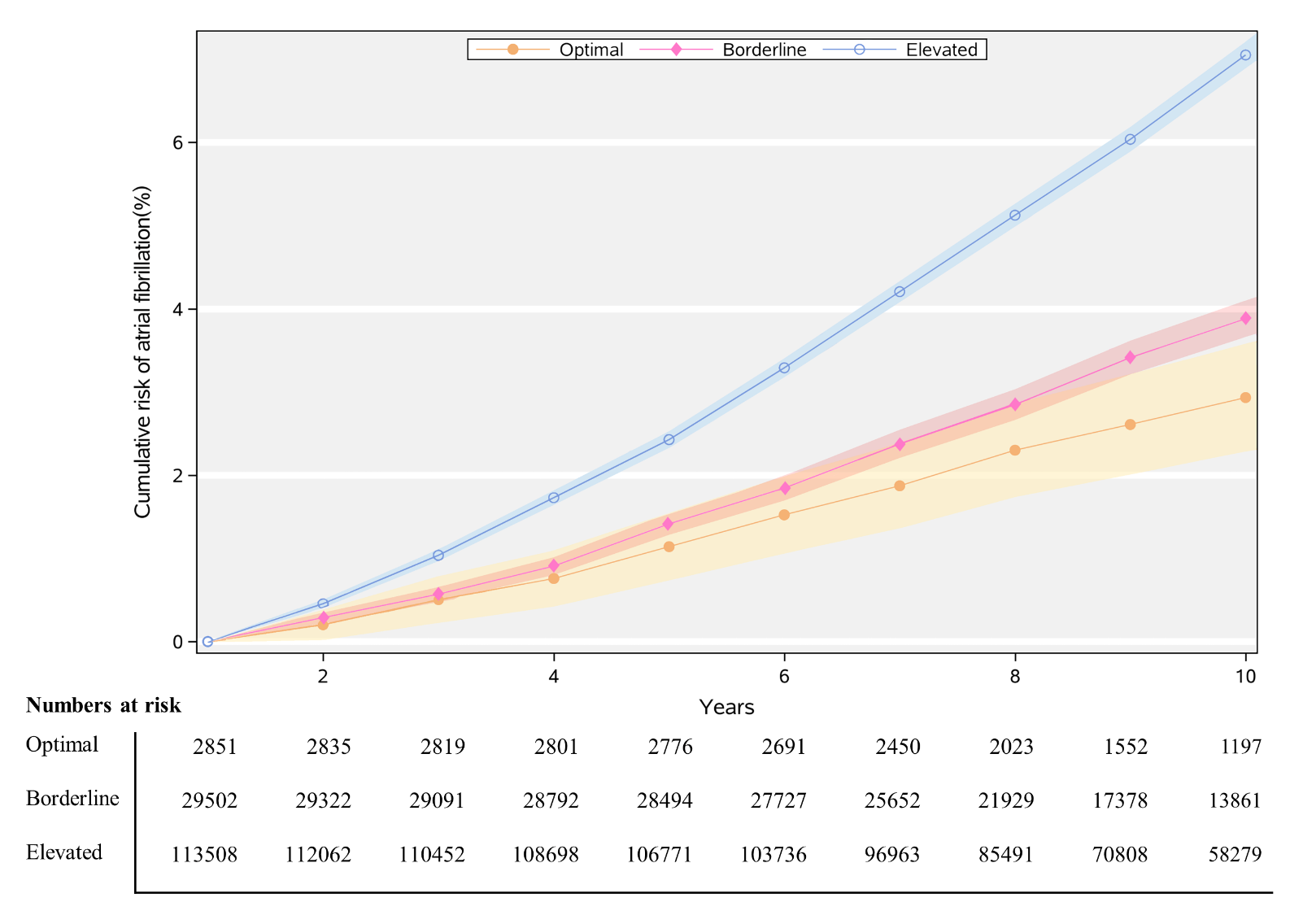


**Figure S2.** Cumulative risk (%) for atrial fibrillation according to risk factor burdens (optimal, borderline, or elevated) at the index age of 65 years. Shading = 95% CI. Participants entered the study sample between ages 65 and < 70 years; therefore, the number at risk increased from age 65 years to < 70 years.


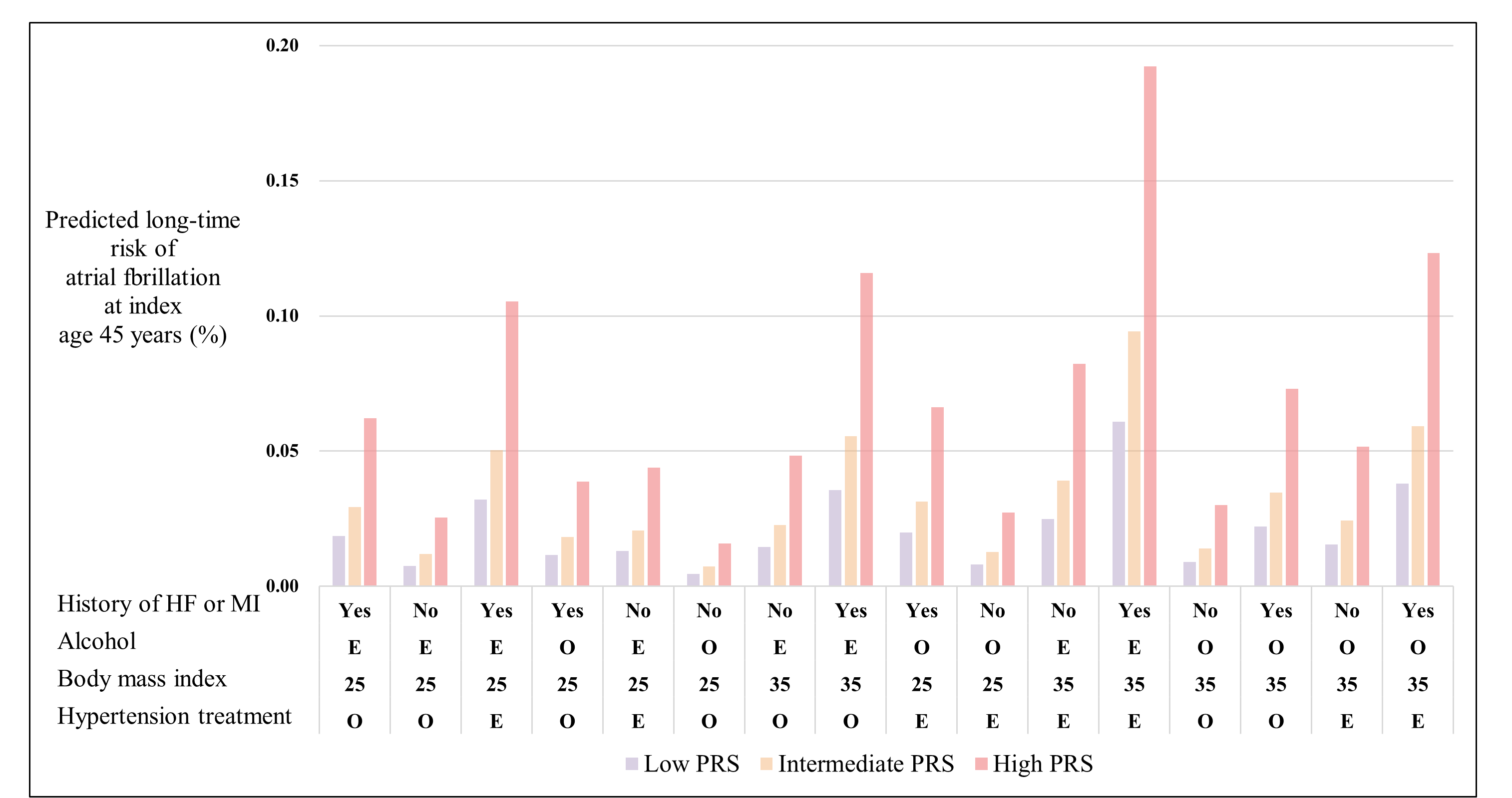


**Figure S3.** Predicted 10-year risk (%) of atrial fibrillation at index age 45 years across 16 risk profiles, in men with different polygenic risk score (low, intermediate, or high).


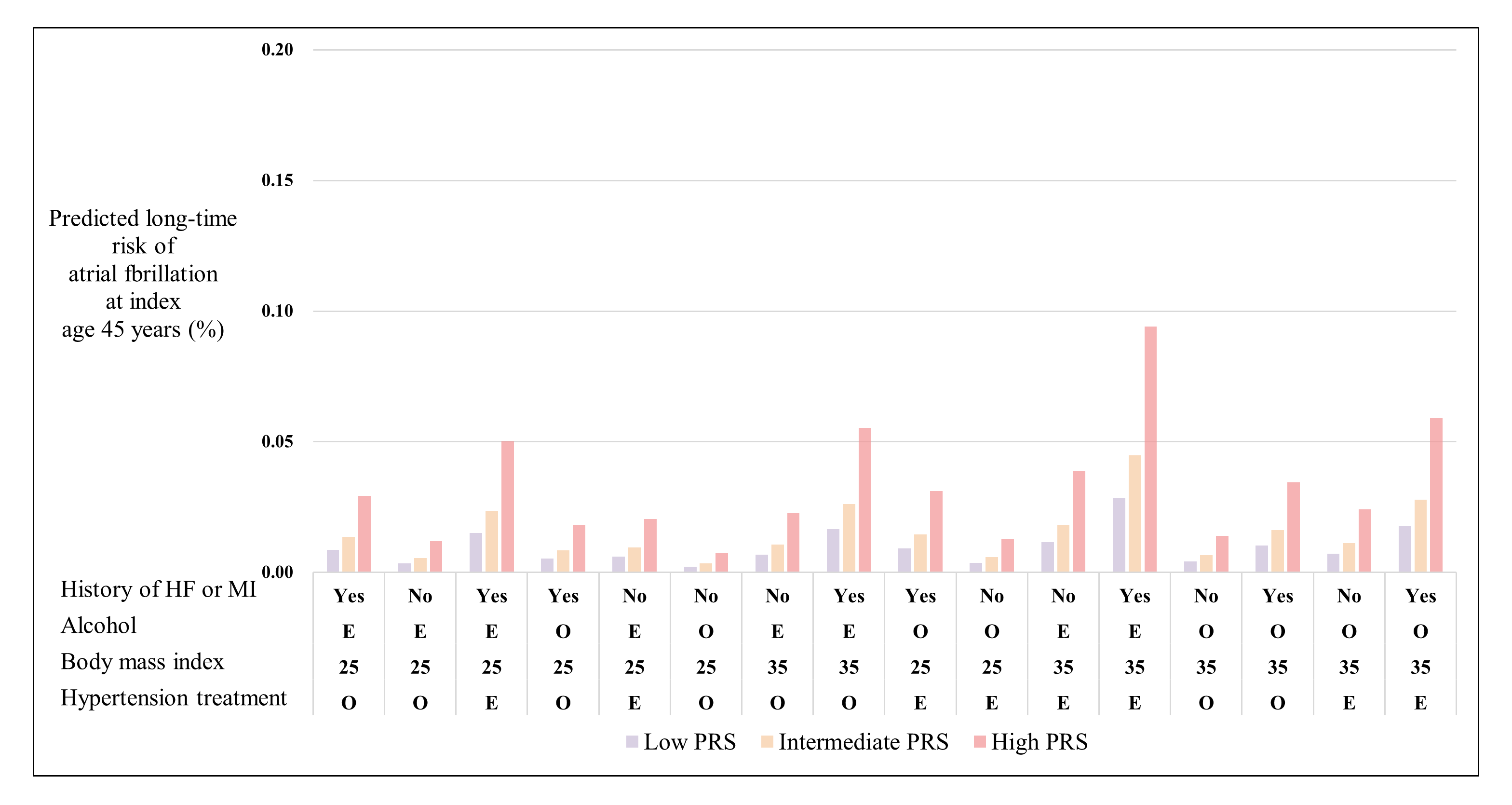


**Figure S4.** Predicted 10-year risk (%) of atrial fibrillation at index age 45 years across 16 risk profiles, in women with different polygenic risk score (low, intermediate, or high).


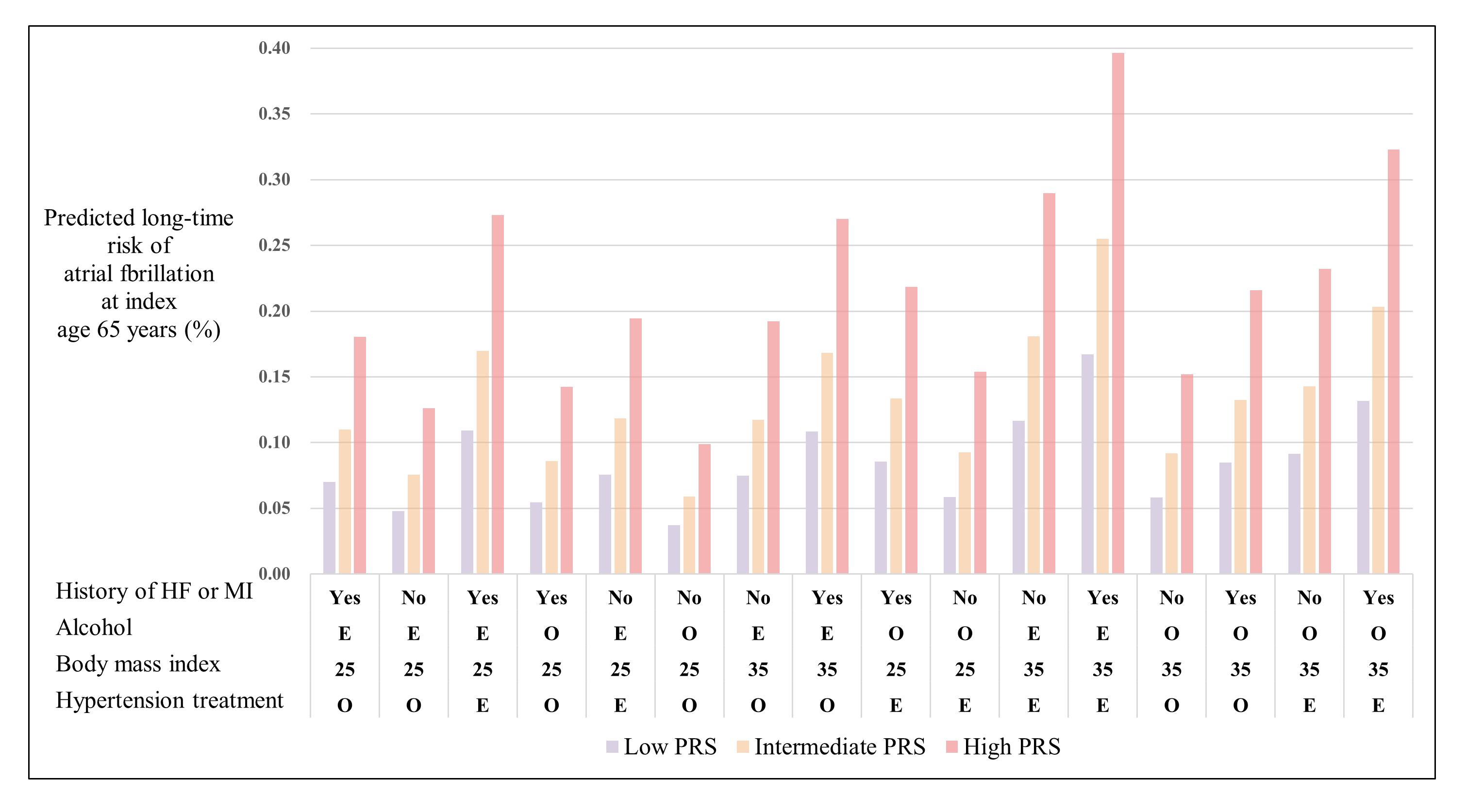


**Figure S5.** Predicted 10-year risk (%) of atrial fibrillation at index age of 65 years across 16 risk profiles, in men with different polygenic risk score (low, intermediate, or high).


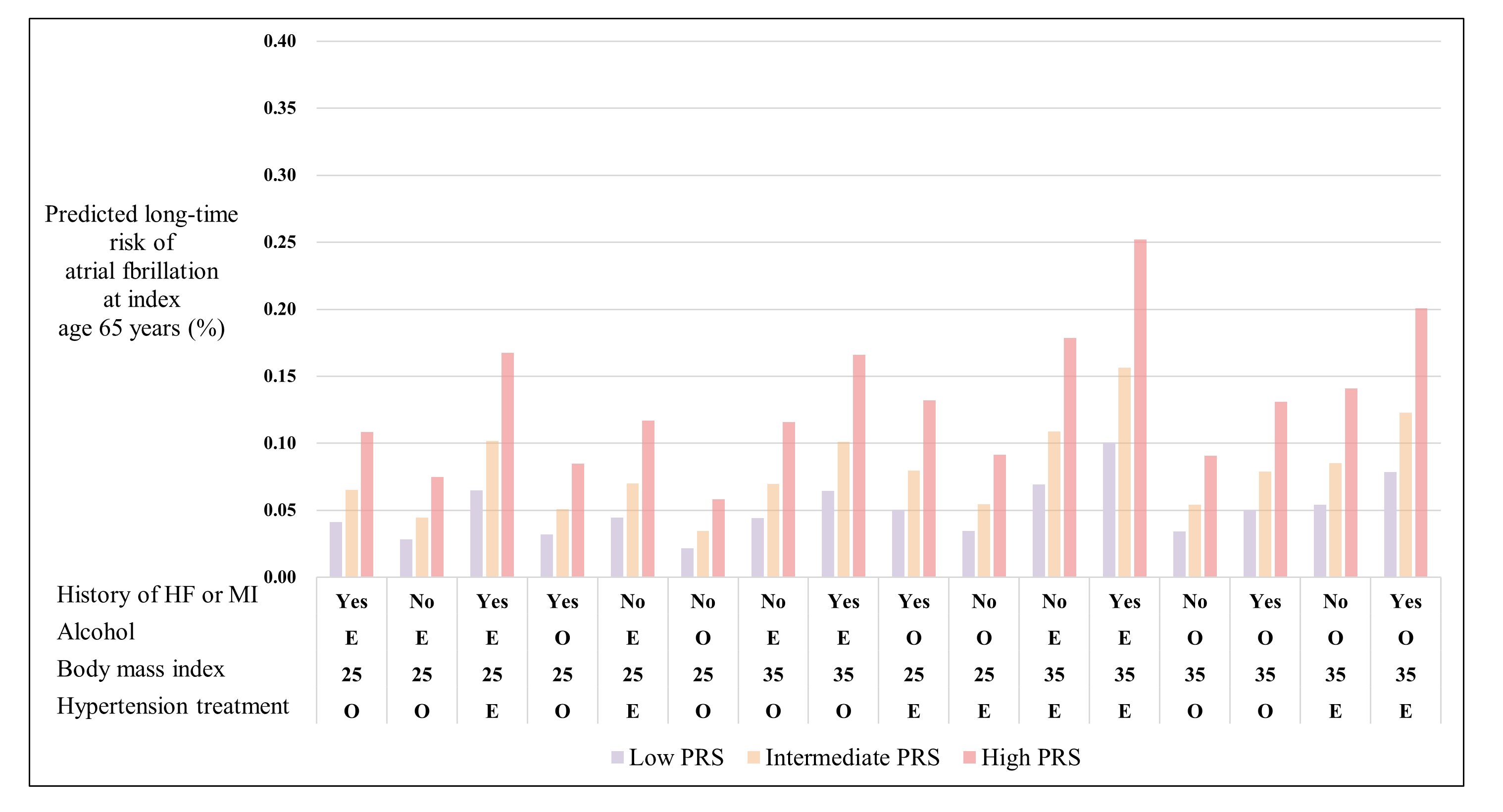


**Figure S6.** Predicted 10-year risk (%) of atrial fibrillation at index age of 65 years across 16 risk profiles, in women with different polygenic risk score (low, intermediate, or high).

**Figure S7.** Calibration plot of the prediction model for predicting 10-year risk at index age of 45 years


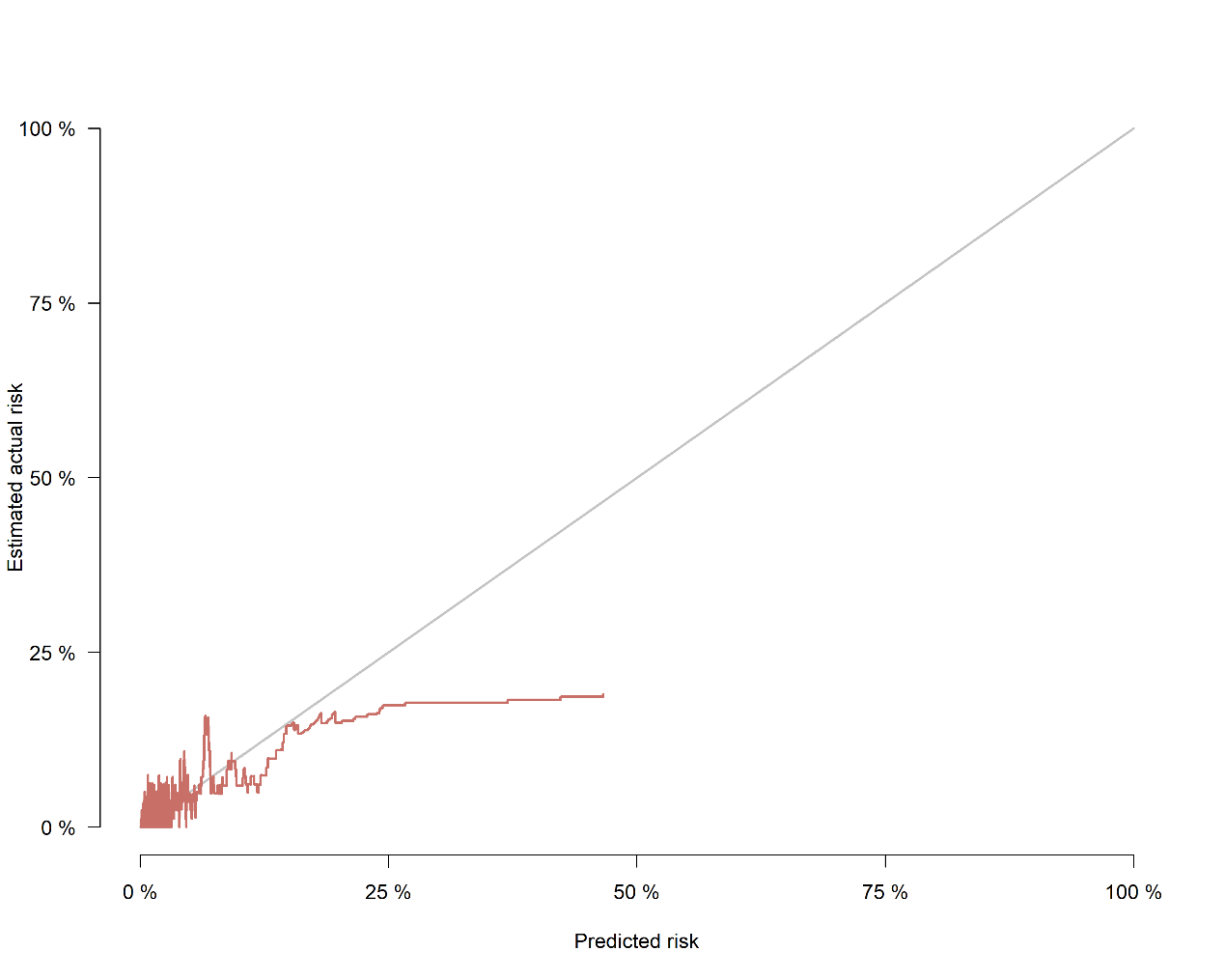


Calibration was good, although the lifetime risk is overestimated for participants in the highest 10-year risk group.

The C index for 10-year risk at index age of 45 years after adjusting for competing risk of death is 73.5% (95% CI: 71.7% to 75.2%).

**Figure S8**. Calibration plot of the prediction model for predicting 10-year risk at index age 55 years


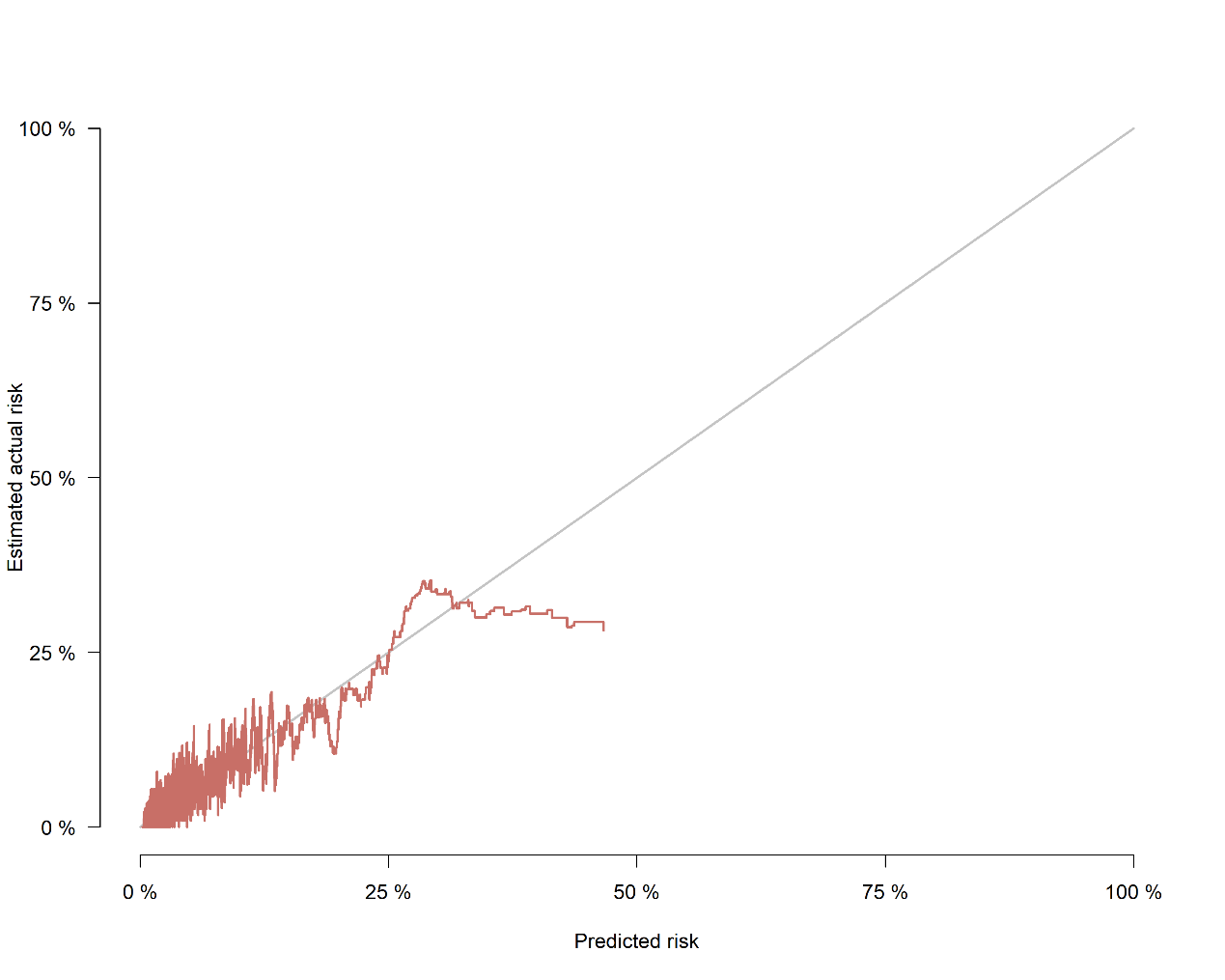


Calibration was good, although the 10-year risk is slightly underestimated for moderate 10-year

risk groups and overestimated for participants in the highest 10-year risk group.

The C index for 10-year risk at index age 55 years after adjusting for competing risk of death is 71.0% (95% CI: 70.1% to 71.9%).

**Figure S9.** Calibration plot of the prediction model for predicting 10-year risk at index age 65 years


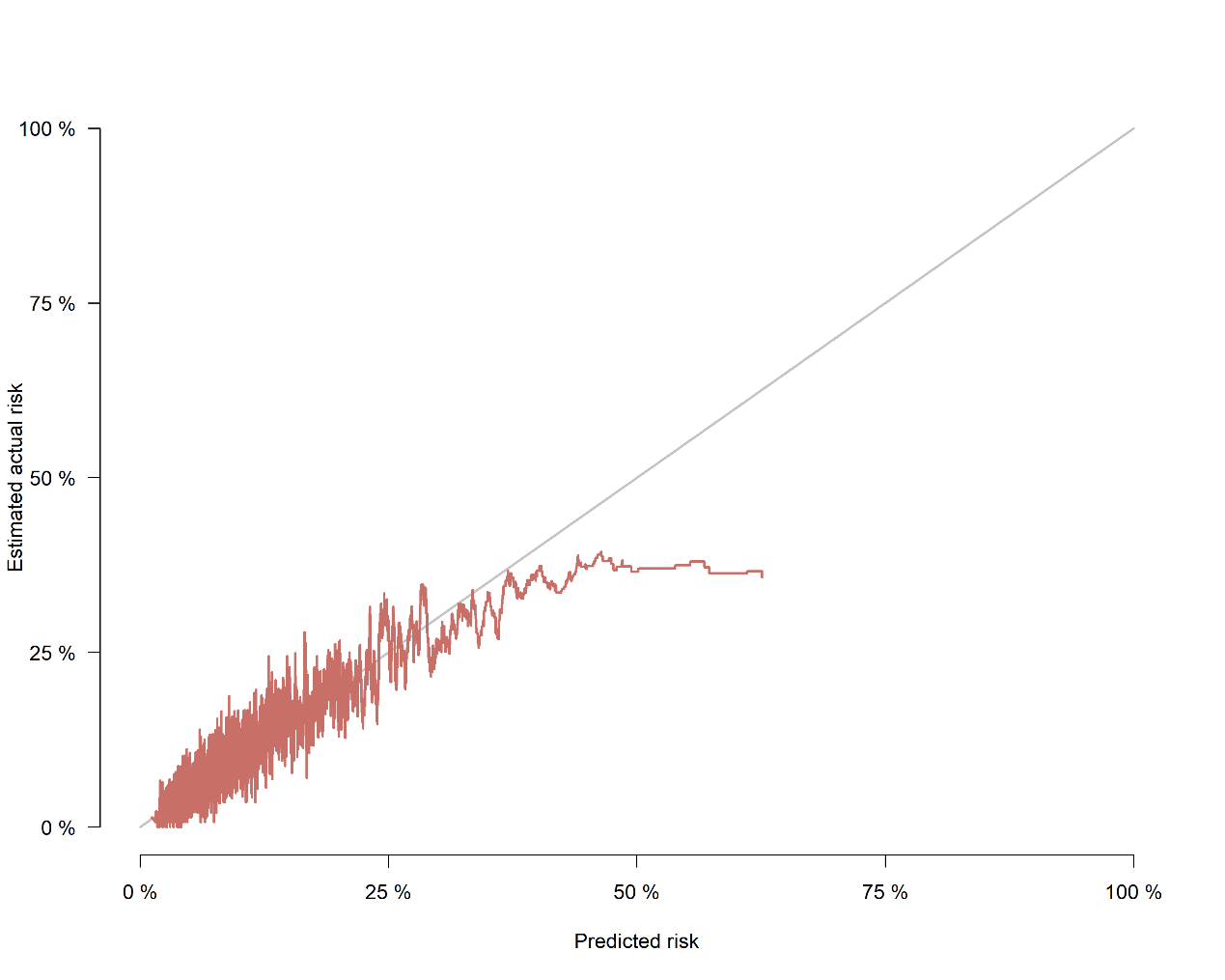


Calibration was good, although the 10-year risk is slightly overestimated for participants in the highest 10-year risk group.

The C index for 10-year risk at index age of 65 years after adjusting for competing risk of death is 67.1% (95% CI: 66.2% to 68.1%).
